# Inclusion of a retroviral protease enhances the immunogenicity of VLP-forming mRNA vaccines against HIV-1 or SARS-CoV-2 in mice

**DOI:** 10.1126/scitranslmed.adt9576

**Published:** 2025-04-30

**Authors:** Peng Zhang, Mamta Singh, Vada A. Becker, Jacob Croft, Yaroslav Tsybovsky, Vinay Gopan, Yuna Seo, Qingbo Liu, Denise Rogers, Huiyi Miao, Yin Lin, Daniel Rogan, Courtney Shields, Sayda M. Elbashir, Samantha Calabrese, Isabella Renzi, Vladimir Preznyak, Elizabeth Narayanan, Guillaume Stewart-Jones, Sunny Himansu, Mark Connors, Kelly Lee, Andrea Carfi, Paolo Lusso

**Affiliations:** 1 Laboratory of Immunoregulation, National Institute of Allergy and Infectious Diseases, NIH, Bethesda, MD 20892, USA; 2 Department of Medicinal Chemistry, University of Washington, Seattle, WA 98195, USA; 3 Moderna Inc., Cambridge, MA 02139, USA; 4 Electron Microscopy Laboratory, Cancer Research Technology Program, Leidos Biomedical Research, Frederick National Laboratory for Cancer Research, Frederick, MD 21701, USA.

## Abstract

Messenger RNA (mRNA) has emerged as a highly effective and versatile platform for vaccine delivery. We previously designed a virus-like particle (VLP)-forming *env-gag* mRNA vaccine against human immunodeficiency virus (HIV)-1 that elicited envelope-specific neutralizing antibodies and protection from heterologous simian-human immunodeficiency virus (SHIV) infection in rhesus macaques. Here, we introduce a key technological advance to this platform by inclusion of mRNA encoding a retroviral protease to process Gag and produce mature VLPs. Appropriately dosed and timed expression of the protease was achieved using a full-length *gag-pol* mRNA transcript. Addition of *gag-pol* mRNA to an HIV-1 *env-gag* mRNA vaccine resulted in enhanced titers of envelope trimer-binding and neutralizing antibodies in a mouse model. Analogous results were obtained with a hybrid Gag-based, VLP-forming severe acute respiratory syndrome coronavirus 2 (SARS-CoV-2) mRNA vaccine expressing an engineered spike protein. Thus, inclusion of a retroviral protease can increase the immunogenicity of Gag-based, VLP-forming mRNA vaccines against human pathogens.

## One Sentence Summary

Addition of a retroviral protease to cleave Gag and generate mature virus-like particles improves mRNA vaccines against HIV-1 or SARS-CoV-2.

## Editor’s Summary

Since the COVID-19 pandemic, mRNA vaccines have taken the realm of infectious disease research by storm. However, their relatively poor durability leaves room for improvement. Here, Zhang *et al.* optimized mRNA vaccines that generate virus-like particles (VLPs) bearing either the HIV-1 envelope or the SARS-CoV-2 spike protein. This optimized platform included a retroviral protease, which enabled the production of mature VLPs with enhanced immunogenicity compared with their immature precursors. The authors found that both HIV-1 and SARS-CoV-2 vaccines including the retroviral protease elicited higher binding and pseudovirus-neutralizing antibodies in mice. These data highlight the promise of VLP-forming mRNA vaccines and provide a means of optimizing such vaccines. –Courtney Malo

## INTRODUCTION

The development of a vaccine capable of inducing protective immunity against human immunodeficiency virus type 1 (HIV-1) continues to represent a priority in the global efforts to terminate the acquired immunodeficiency syndrome (AIDS) pandemic. After infection, HIV-1 rapidly integrates its proviral genome into the host chromosomes to establish a lifelong persistent infection that, in the absence of treatment, leads to progressive immune suppression, opportunistic infections and neoplasias, and death (1). This implies that a protective vaccine must prevent even a single integration event of replication-competent virus and, therefore, must confer sterilizing immunity, which requires the production of broadly neutralizing antibodies (bNAbs) (2, 3). An important proof-of-principle of the feasibility of a protective vaccine was obtained with passive protection studies in nonhuman primate models infused with bNAbs directed against conserved sites of HIV-1 vulnerability (3, 4). In spite of intensive efforts over the past three decades, however, a vaccine capable of inducing bNAbs has yet to be developed. This shortcoming is largely due to the vast repertoire of immune-evasion tactics enacted by the HIV-1 envelope (Env) glycoproteins, which includes a marked antigenic variability, an uncommon conformational flexibility and a dense glycan shield covering almost the entire exposed surface of the Env spike (2, 4, 5). In particular, the extensive and highly heterogeneous glycosylation of the HIV-1 Env represents a major challenge that has to be carefully considered in the design of an effective vaccine. The remarkable immune evasion skillset of HIV-1 can also explain why even natural infection induces the production of bNAbs only in a relatively small proportion of individuals and only after months or years of persistent antigenic stimulation (4).

A wide variety of vaccine approaches have been evaluated against HIV-1 or its simian counterpart, simian immunodeficiency virus (SIV). Subunit vaccines based on monomeric gp120 or soluble stabilized Env trimers (SOSIP) were shown to elicit predominantly autologous neutralizing antibodies targeting isolate-restricted epitopes in the variable loops or glycan holes but little, if any, neutralization breadth (6–10). In contrast, epitope-focused approaches targeting the fusion peptide in gp41 based on synthetic peptide priming, followed by soluble trimer boosting, have induced some degree of neutralization breadth (11, 12). Other vaccine strategies based on prime-boost schemes, including but not limited to ALVAC plus pentavalent gp120 (13), adenovirus (Ad) 26-expressed mosaic Env and Gag-Pol antigens (14), DNA plus gp120 (15), DNA plus modified vaccinia Ankara (MVA) plus SOSIP trimers (16), MVA plus multimeric Env gp145 (17), MVA plus cyclically permuted trimeric gp120 (18), or a combination of heterologous viral vectors and SOSIP trimers (19) all have reported some degree of protection in nonhuman primate models. A distinct approach, based on sequential schooling of bNAb-producing B-cell lineages has yielded encouraging initial results in recruiting the appropriate precursor B cells (20–24). However, none of the approaches tested so far has been able to progress past the initial step of recruitment and early expansion of B cells expressing germline bNAb precursors. Only a single human clinical trial, the RV144 study performed in Thailand with poxvirus-expressed HIV-1 Gag, protease (Pro), and Env followed by recombinant gp120, has reported a limited and transient protective activity, with a risk reduction of approximately 30% (25), although this result was not subsequently reproduced (26). Altogether, these results confirm the unprecedented challenges inherent to the elicitation of protective immunity against HIV-1 that conventional vaccine approaches have proven unable to meet.

Intensive efforts are currently focused on optimizing germinal center (GC) responses to vaccines in secondary lymphoid organs through Env multimerization either on nanoparticles or on virus-like particles (VLPs) (24, 27–30). The Env proteins presented on particulate immunogens, especially when expressed as full-length, membrane-bound gp120/gp41 heterotrimers, closely mimic the structure and antigenicity of the native Env spikes. HIV-1 vaccines based on VLPs were found to induce strong antibody responses in preclinical models (31–35). Besides preserving the native conformation of Env, nanoparticles and VLPs provide additional advantages over recombinant Env proteins, as large-size multimeric antigens induce more effective responses by both the innate and adaptive arms of the immune system (36, 37). One mechanism for such increased responses is a more efficient uptake and processing by antigen-presenting cells (38).

We recently developed and tested in preclinical models a vaccine platform that employs mRNA as a vehicle to produce HIV-1 Env-expressing VLPs in vivo (39). In this platform, VLP formation was achieved by the addition of SIV Gag, which was co-formulated in LNPs with Env mRNA to ensure co-expression by the same transfected cells. Endogenous expression by host cells through mRNA guarantees the production of membrane-anchored Env proteins bearing native glycosylation. We initially tested this platform in mice and found that an *env-gag* mRNA vaccine was more effective than *env* mRNA alone in inducing neutralizing antibodies (39). A similar platform was adapted for a hybrid SARS-CoV-2 vaccine and yielded similar results (40). Furthermore, in rhesus macaques, priming with a transmitted-founder clade-B *env* mRNA lacking the critical N276 glycan, followed by multiple booster immunizations with glycan-repaired autologous and subsequently bivalent heterologous *envs* (clades A and C), all co-formulated with *gag* mRNA, was highly immunogenic and eventually led to the induction of heterologous tier-2 NAbs accompanied by robust anti-Env CD4^+^ T cell responses. Immunized animals were protected from repeated low-dose mucosal challenges with a difficult-to-neutralize heterologous tier-2 simian-human immunodeficiency virus, or SHIV (SHIV AD8) (39). These results suggest that immunization with membrane-anchored Env, the use of mRNA as a vector, the formation of VLPs, and an intensive multiclade heterologous boosting schedule may all have contributed to the success of this vaccine platform.

In this study, we aimed at further optimizing the VLP-forming *env-gag* mRNA vaccine platform by introducing Pro in order to fully process Gag and produce mature, rather than immature VLPs. When viral particles or VLPs are produced in the absence of Pro, they resemble immature, non-infectious virions, similar to those produced in the presence of protease inhibitors (41). These immature virions contain a stiff and amorphous Gag lattice that interacts with the gp41 cytoplasmic tail and prevents the clustering of Env spikes by maintaining them sparsely distributed across the particle surface. Conversely, introduction of a functional Pro, which cleaves the matrix protein (MA) from the capsid protein (CA), allows for the untethered movement of the Env spikes on the particle surface resulting in their coalescence into discrete clusters (41–44). Since Env clustering on mature HIV-1 particles is believed to lead to more efficient B cell stimulation through cross-linking of the B cell receptor, as well as enhanced antigen uptake, processing and presentation by antigen-presenting cells, we reasoned that immunization with mature, rather than immature VLPs might improve vaccine immunogenicity. Thus, we performed an extensive search for the appropriate conditions for Pro expression using mRNA, which poses a series of technical challenges due to the unique mechanism of Pro translation during the retroviral life cycle (45). An optimized mRNA construct was selected, encompassing the full-length Gag-Pol transcript. To evaluate the in vivo functionality of such construct and the potential benefit of Pro addition to a VLP-forming HIV-1 *env-gag* mRNA vaccine, we compared immunization with different mRNA formulations in mouse model. Moreover, an analogous design was applied to a VLP-forming mRNA vaccine against SARS-CoV2 containing a C-terminally engineered spike protein (40). In both immunization models, the addition of Pro resulted in improved trimer-binding and neutralizing antibody responses. These results indicate that inclusion of Pro may provide an optimized platform for Gag-based VLP-forming vaccines.

## RESULTS

### Stably transduced HEK 293T clones expressing native HIV-1 Env trimers are suitable for VLP production

To obtain a reproducible, high-throughput system for the production of retroviral Gag-based VLPs in vitro, we generated stable cell clones with constitutively high expression of the HIV-1 Env using a previously described retroviral transduction method (46). The abundance of Env expression on the cellular membrane is a major determinant of the efficiency of VLP production and is affected by specific domains in the cytoplasmic tail of the gp41 Env subunit, especially the C-terminal endocytosis domain and the lentivirus lytic peptide 2 (47–49). As an initial step, we therefore aimed at selecting the minimal gp41 cytoplasmic tail truncation yielding the highest amount of surface membrane expression while retaining a native-like antigenic profile. Truncations of different length (at aa. 745, 754, 764 or 770) were made in the gp41 cytoplasmic tail of a clade-B, tier-2 HIV-1 Env (WITO 4160.27_G153E), and the truncated mutants were expressed in HEK 293T cells and tested for their recognition by a large panel of bNAbs and non-neutralizing antibodies (nNAbs) by flow cytometry. Env expression was higher in the two shorter mutants compared with the longer mutants, with the 1–745 construct, truncated immediately downstream of the Kennedy epitope (aa. 722–743), displaying the highest expression, as determined using the reference antibody 2G12; the 1–745 mutant also showed the best antigenic profile, with higher reactivity with bNAbs such as VRC01, PGT145, and CH01 without a corresponding increase in reactivity with nNAbs such as b13 and 41.2d (fig. S1). Based on these results, the 1–745 cytoplasmic tail truncation was selected as the Env truncation for the establishment of stable Env-expressing cell clones.

To generate stably transfected cell lines constitutively expressing high amounts of HIV-1 Env, lentiviral expression plasmids were synthesized by subcloning DNA from two biologically distinct clones of the WITO Env (WITO.27_1.745, an open, tier-1b Env, and WITO.27_G153E_1–745, a closed tier-2 Env) into the pLenti-III-HA vector (pLenti-Env). Lentiviral particles were produced by co-transfecting HEK 293T/17 cells with three plasmids (pLenti-Env, a packaging plasmid, psPAX2, and a vesicular stomatitis virus (VSV) G protein-expressing plasmid). Following transduction, the HIV-1 Env DNA became integrated into the genome of HEK 293T cells. Transduced cells were initially expanded in bulk in the presence of the selection antibiotic puromycin and then single-cell sorted by gating on high Env expression, as determined by flow cytometry. Individual clones were expanded and tested for Env expression over time. Env expression remained stably elevated in all the clones tested, with no apparent modification over time. Two clones with the highest Env expression (clone A6 for WITO.27_1.745 and clone 2A11 for WITO.27_G153E_1–745) were selected for further studies (fig. S2).

We previously demonstrated that co-transfection of HIV-1 *env* and SIV *gag* mRNA leads to the production and extracellular release of Env-rich VLPs (39). To evaluate the efficiency of VLP production in cells with a high constitutive Env expression, a ΔEnv HIV-1 backbone plasmid (pSG3) was transfected into 293T-WITO.2A11 cells or, as a control, into regular 293T cells simultaneously co-transfected with the WITO.27_G153E_1–745 Env plasmid. VLP formation was assessed by measuring the amount of captured magnetic beads-mediated particle capture using the trimer-specific bNAb PG16. The efficiency of extracellular VLP production was higher in the stable cell clone compared to the transiently Env-transfected cells (fig. S2). These results demonstrated that the use of stable Env-expressing cells can provide a highly efficient system to produce HIV-1 VLPs while averting the need for transient Env transfection.

### Addition of mRNA encoding Pro leads to inefficient production of mature VLPs

**Fig. 1.**
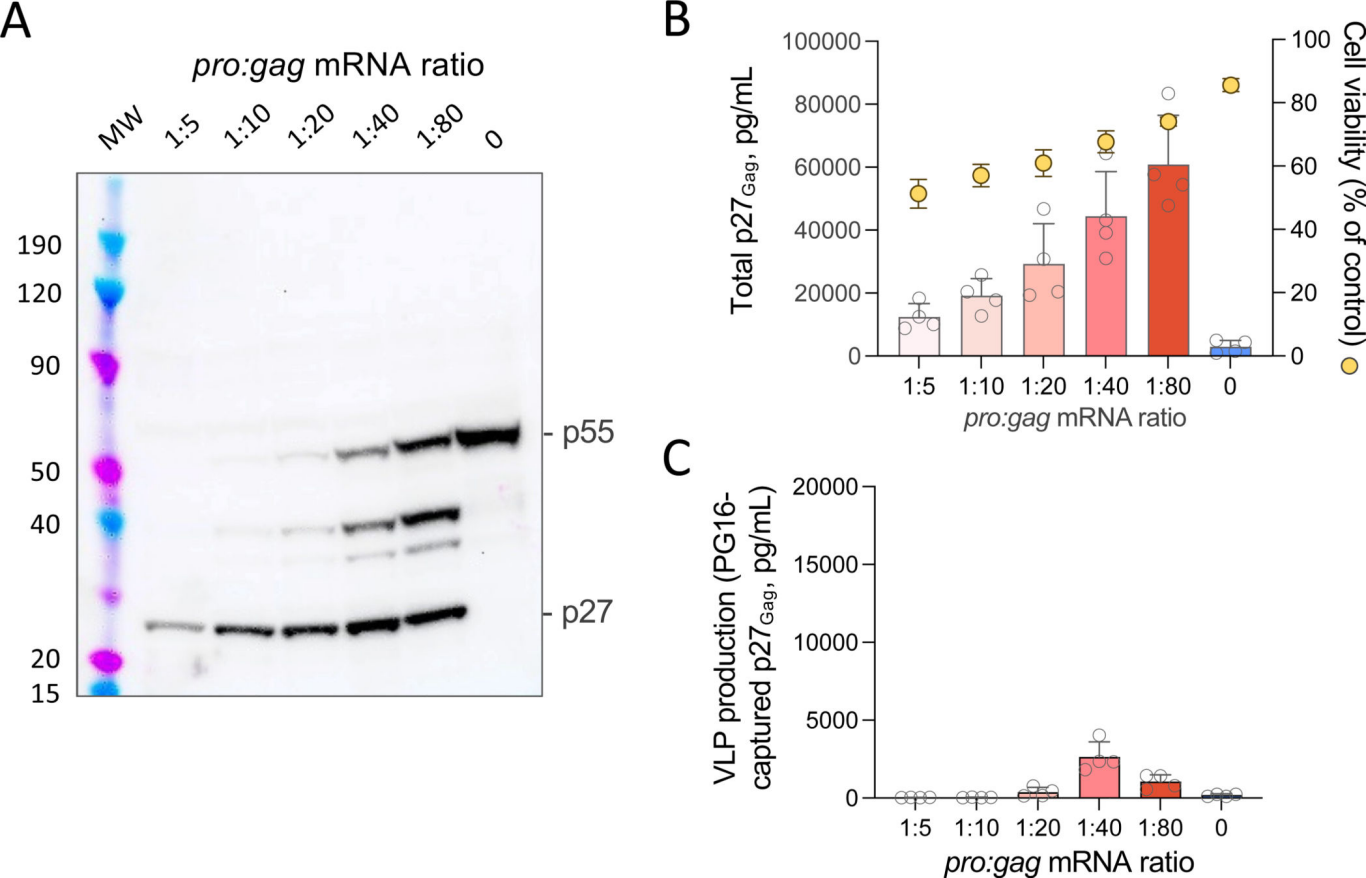
Addition of mRNA expressing a retroviral protease results in efficient Gag processing but inefficient extracellular VLP production. (**A**) Immunoblot analysis of Gag p55 processing in stably Env-expressing HEK293T cells co-transfected with SIV *gag* mRNA (2 μg per reaction) and *pro* mRNA at the ratios indicated on the top of the image. The blot was visualized using an anti-SIV Gag antibody that recognizes both uncleaved (p55) and cleaved (p27) SIV Gag. MW, molecular weight. (**B**) Quantification of total Gag p27 concentration in the culture supernatant of transfected cells. The amount of total Gag p27 (left *y* axis) was quantified after 48 hours of transfection using an ELISA specific for cleaved p27. Cell viability (right *y* axis) was measured at 48 hours by flow cytometry using a live-dead dye. (**C**) Quantification of VLP-associated Gag p27 captured from the culture supernatants of cells transfected as in (B) collected at 48 hours post-transfection using the trimer-specific human bNAb PG16 bound to magnetic beads. The amount of Gag p27 was quantified using the same ELISA as in (B). Data in (B and C) are presented as the mean (± standard error of the mean) from two technical replicates each from two representative experiments.

Although co-expression of Env and Gag leads to the efficient production of Env-rich VLPs (39), in the absence of the viral Pro, such VLPs remain immature as they contain exclusively unprocessed Gag (p55). In contrast, when Gag is processed by Pro to yield its cleavage products, matrix (MA), capsid (CA), nucleocapsid (NC) and p6, mature VLPs are produced (41). In an initial attempt to generate mature HIV-1 VLPs using mRNA, we generated an mRNA corresponding to the entire coding sequence of Pro from SIVmac239 and co-transfected it with SIV *gag* mRNA into the Env-expressing 293T-WITO.2A11 cell clone at different *pro:gag* mRNA ratios (from 1:5 to 1:80, Wt:Wt). As discussed in our original report (39), the rationale for generating hybrid VLPs containing HIV-1 Env and SIV Gag, stemmed from the need to validate our vaccine in a suitable pre-clinical SHIV challenge model in rhesus macaques. A near complete processing of the Gag precursor, p55, was seen by immunoblot at the highest Pro concentrations (1:5 to 1:20), whereas an increasing fraction of p55 remained uncleaved at 1:40 and 1:80 ratios (Fig. 1A). However, higher Pro concentrations caused a reduction of cell viability and, as a consequence, a drop in the total amount of p27 production (Fig. 1B). When we analyzed the extracellular release of mature VLPs by PG16-mediated capture, VLP production was detected only at the lowest two concentrations of Pro, with only a minor fraction of the total cleaved p27 (less than 1%) being incorporated into nascent pseudovirus particles (Fig. 1C). These results suggest that expression of Pro as a separate gene may lead to an excessive and untimely activation of the enzyme, resulting in enhanced cellular toxicity and premature release of cleaved p27 within the cytoplasm prior to its incorporation into budding particles.

### Pro expression through full-length *gag-pol* transcripts results in the efficient production of mature HIV-1 VLPs

**Fig. 2.**
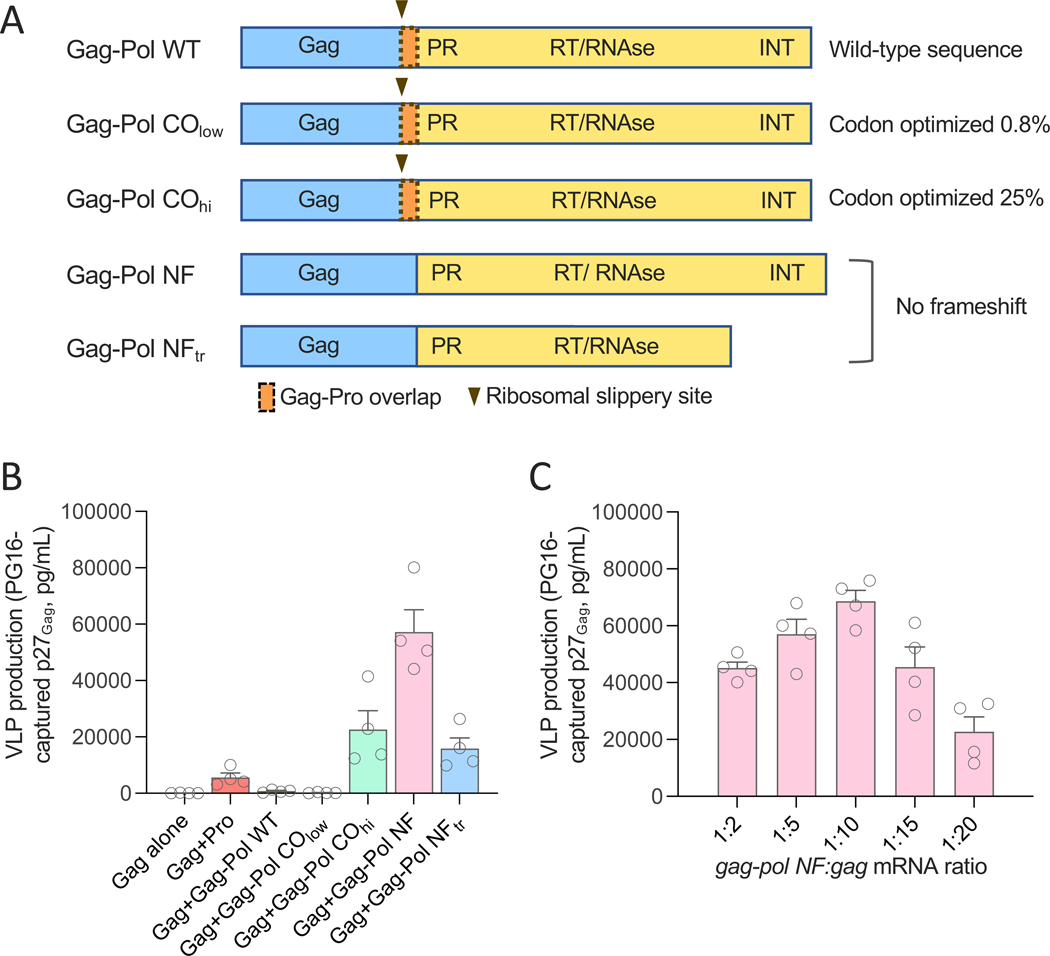
Different *gag-pol* mRNA constructs result in variable expression of the SIV protease and production of mature VLPs. (**A**) Schematic representation of five different SIV *gag-pol* mRNA constructs with or without the ribosomal frameshift mechanism and variable amounts of codon optimization. (**B**) Quantification of extracellular VLPs produced by stably Env-expressing HEK293T cells co-transfected with SIV *gag* mRNA (1 μg per reaction) and different *gag-pol* mRNA constructs (1 μg per reaction) or controls. VLPs were captured from the culture supernatants 48 hours after transfection using the trimer-specific human bNAb PG16 bound to magnetic beads. The amount of VLP-associated Gag p27 was quantified using an ELISA specific for cleaved SIV p27. (**C**) Quantification of VLP-associated Gag p27 captured from the culture supernatants of cells co-transfected with *gag* mRNA and decreasing amounts of *gag-pol NF* mRNA at the indicated *gag-pol:gag* mRNA ratios; samples were collected at 48 hours post-transfection. Data in (B and C) are presented as the mean (± standard error of the mean) from two technical replicates each from two representative experiments.

To overcome the drawbacks associated with excessive and premature Pro activation, we attempted to mimic the physiological mechanism that takes place in the course of HIV-1 infection, wherein Pro is transcribed as part of the long *gag-pol* mRNA transcript and subsequently translated into protein from a different reading frame through a ribosomal frame-shift mechanism (45). Five different *gag-pol* mRNA designs, based on the SIVmac239 sequence, were synthesized to identify the most suitable to induce appropriately dosed and timed expression of Pro for VLP formation (Fig. 2A). Three of the constructs maintained the ribosomal frameshift mechanism and, therefore, were not fully codon-optimized to avoid altering the frameshift-recognition and *gag-pro* gene-overlap sites: 1. Gag-Pol WT, containing the full-length wild-type (WT) *gag-pol* sequence with no codon optimization; 2. Gag-Pol CO_low_, featuring a very limited codon optimization (0.8%) to enhance protein translation; and 3. Gag-Pol CO_hi_, featuring a more substantial codon optimization (25%). Two constructs did not contain the ribosomal slippery site and gene overlaps, and thus could safely be codon optimized: 4. Gag-Pol NF, containing the full-length *gag-pro-pol-int* transcript with all genes in the same reading frame; and 5. Gag-Pol NF_tr_, containing the same sequence as #4 but truncated at aa. 1157 (aa. 767 of Pol). Only minimal expression of Gag protein was detected when the *gag-pol* mRNA constructs were transfected alone into 293T-WITO.2A11 cells (fig. S3). We reasoned that the long *gag-pol* mRNA transcript induced the translation of only a limited amount of Gag and thus we decided to co-transfect each *gag-pol* mRNA construct together with SIV *gag* mRNA. The highest amount of extracellular VLP production, measured by the amount of PG16-capturable p27, was detected with the full-length frameshift-free construct, Gag-Pol NF (no. 4), followed by the partially codon-optimized Gag-Pol CO_hi_ (no. 3) and the truncated Gag-Pol NF_tr_ (no. 5) (Fig. 2B). Based on these results, the codon-optimized Gag-Pol NF construct was selected for further studies.

To define the optimal amount of *gag-pol* mRNA for VLP production, we co-transfected 293T-WITO.2A11 cells with a fixed amount of *gag* mRNA (1 μg) and decreasing amounts of *gag-pol NF* mRNA (1:2 to 1:20 *gag-pol:gag* ratio). The efficiency of extracellular VLP production showed a gaussian distribution with a peak at the ratio of 1:10 (*gag-pol:gag*) (Fig. 2C). Based on these results, we concluded that co-transfection of *gag* with codon-optimized *gag-pol NF* mRNA at a 1:10 ratio provides optimized conditions for mRNA-directed production of mature VLPs.

### Immature and mature HIV-1 VLPs display different morphological features

**Fig. 3.**
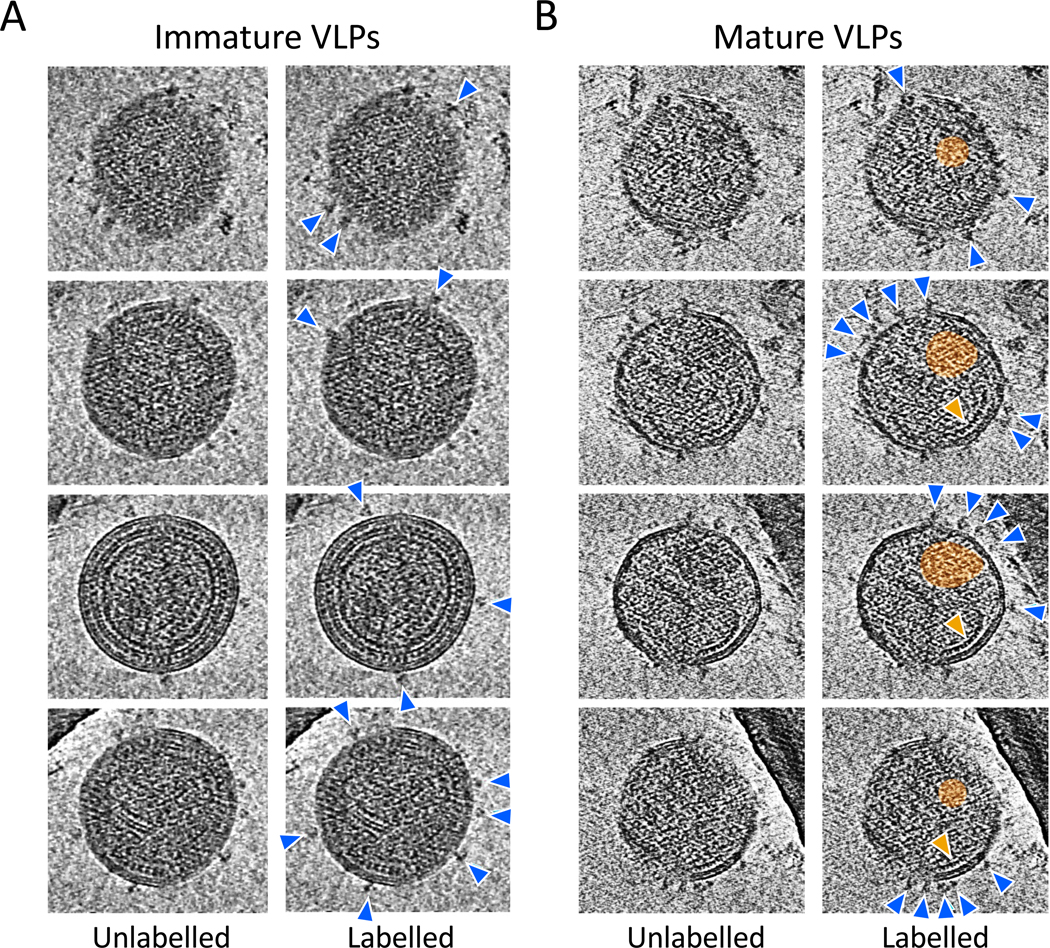
mRNA-expressed immature and mature HIV-1 VLPs display different ultrastructural features. (**A**) Cryo-electron tomogram of an immature virus-like particle in which the internal Gag polyprotein lattice is clearly visible and few copies of Env (blue arrows, right column) are displayed on the VLP surface. Serial computational sections through the 3D tomogram are shown from top to bottom. Scale bar, 100 nm. (**B**) Cryo-electron tomogram of a mature virus-like particle that has undergone proteolytic cleavage of the internal Gag polyprotein. Serial computational sections through the 3D tomogram are shown from top to bottom. Left column, raw tomogram; right column annotated to indicate Env (blue arrows) and Gag core (orange shaded), as well as a second partially formed core (orange arrow). Scale bar, 100 nm.

The morphological features of mature and immature VLPs produced in vitro upon mRNA transfection of *env* and *gag* mRNA with or without *gag-pol* mRNA into 293-T cells were characterized by cryo-electron tomography (cryoET). Both immature (no *gag-pol* mRNA) and mature (with *gag-pol* mRNA) VLPs presented with particle diameters of approximately 140 to 160 nm. Immature VLPs featured a well-organized, assembled Gag lattice underneath the viral membrane and displayed infrequent Env spikes on their surface, which did not show clustering (Fig. 3A). In contrast, an organized Gag lattice was not observed in mature VLPs, and some particles showed initial core condensation; mature VLPs displayed a higher density of Env spikes, with apparent clustering of 3 to 4 spikes visible on the surface of some particle (Fig. 3B). On average, mature VLPs displayed higher numbers of Env spikes on their surface (mean ± SD: 23 ± 12) compared with immature VLPs (mean ± SD: 16 ± 7), although there was wide variability. Of note, approximately 20% of the particles produced in the presence of *gag-pol* mRNA displayed an immature morphology, indicating incomplete Gag cleavage possibly due to the relatively early harvesting time (48 hours) after transfection.

### Inclusion of Pro enhances the immunogenicity of an HIV-1 *env-gag* mRNA vaccine in mice

**Fig. 4.**
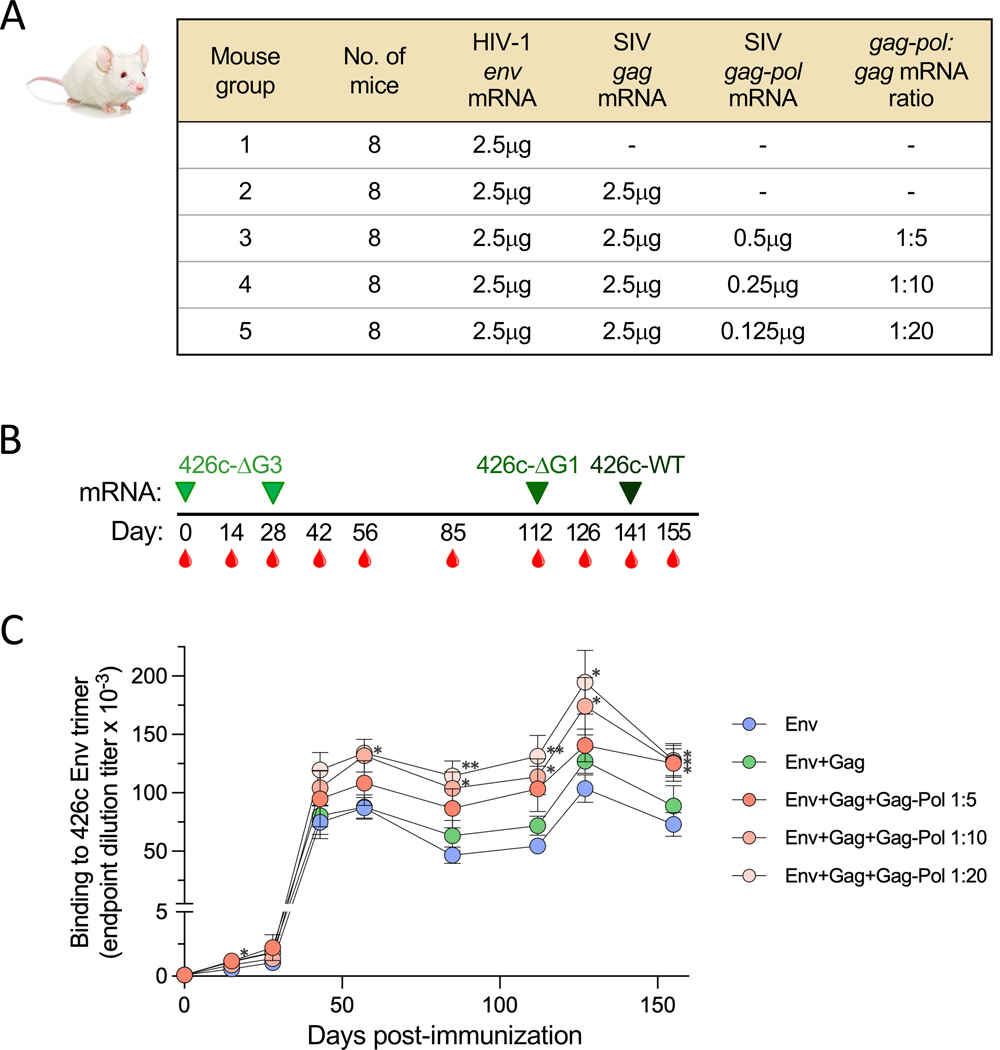
Immunogenicity of HIV-1 mRNA vaccines producing immature or mature VLPs in mice. (**A**) Design of the immunization study. Each vaccine arm included eight wild-type BALB/c mice. Mice were immunized with in LNP-formulated mRNA encoding HIV-1 426c Env and SIV mac239 Gag with or without Gag-Pol NF. (**B**) Schematic time course of immunizations (green arrows) and bleedings (red drops). 426c-ΔG3 is deglycosylated at three positions: N276D, N460D and N463D; 426c-ΔG1 at one position: N276D; 426c-WT is fully glycan repaired. (**C**) Induction of 426c trimer-binding antibodies in the 5 study arms over time was assessed by ELISA. Data are presented as mean values (±SEM) of endpoint titers for each study arm. Statistical comparisons were made by Kruskal-Wallis test followed by post-hoc Dunn’s correction for multiple comparisons with Arm 1. The asterisks indicate significant *p*-values: * < 0.05; ** < 0.01.

Next, to assess in vivo function of the optimized mRNA construct for the expression of the SIV Pro, we designed a pre-clinical immunization study in mice to compare the immunogenicity of mRNA formulations expressing membrane-bound HIV-1 Env either alone or in combination with SIV Gag plus or minus the SIV Gag-Pol NF construct to express Pro. To identify the optimal mRNA formulation for Pro activity in vivo, three different Wt:Wt ratios between *gag-pol* and *gag* mRNA were tested: 1:5, 1:10 and 1:20. Wild-type (WT) BALB/c mice (n=8 per vaccine arm) were immunized with 5 different mRNA regimens (Fig. 4A). Since our objective was to evaluate potential improvements induced by Pro addition, a sub-optimal amount of *env* mRNA was used (2.5 μg/dose). The mice were immunized twice, on days 0 and 28, with an open, germline VRC01-engager Env, 426c-deglyco-3 (ΔG3), in which three critical glycans (N276D, N460D, N463D) that protect the CD4-binding site (CD4-BS) were removed, followed by one immunization, on day 112, with a partially glycan-repaired version of the same Env (426c-deglyco-1 (ΔG1), N276D), and finally by a fourth immunization, on day 141, with the fully glycosylated form of the same Env (426c-WT). The animals were bled once before immunization, every two weeks until day 56, and then every four weeks except on day 126, two weeks after the third immunization (Fig. 4B).

Vaccine responses were first assessed by measuring serum titers of trimer-binding and neutralizing antibodies. Trimer-binding antibodies, measured by end-point dilution immunoassays performed with lectin-captured soluble 426c SOSIP trimers, became detectable at low titers after the first immunization, showed an increase after the second immunization, reaching an initial peak on day 56, and then were boosted after the third immunization; no further increase in titer was seen after the final immunization (Fig. 4C). All three groups of mice receiving Pro-containing regimens developed higher titers than Arm 2; in Arms 4 and 5, the titers were significantly higher than in Arm 1 at multiple time points (p < 0.05 for Arm 4 on days 85, 112, 126 and 155; for Arm 5 on days 14, 56, 126 and 155; p < 0.01 for Arm 4 on days 85 and 112), whereas Arm 3 had a significantly higher response only on day 155 (p = 0.0411), as assessed by Kruskal-Wallis test followed by Dunn’s correction for multiple comparisons (Fig. 4C). The mean area under the curve (AUC) for each vaccine Arm showed the same hierarchy of vaccine responses (table S1).

**Fig. 5.**
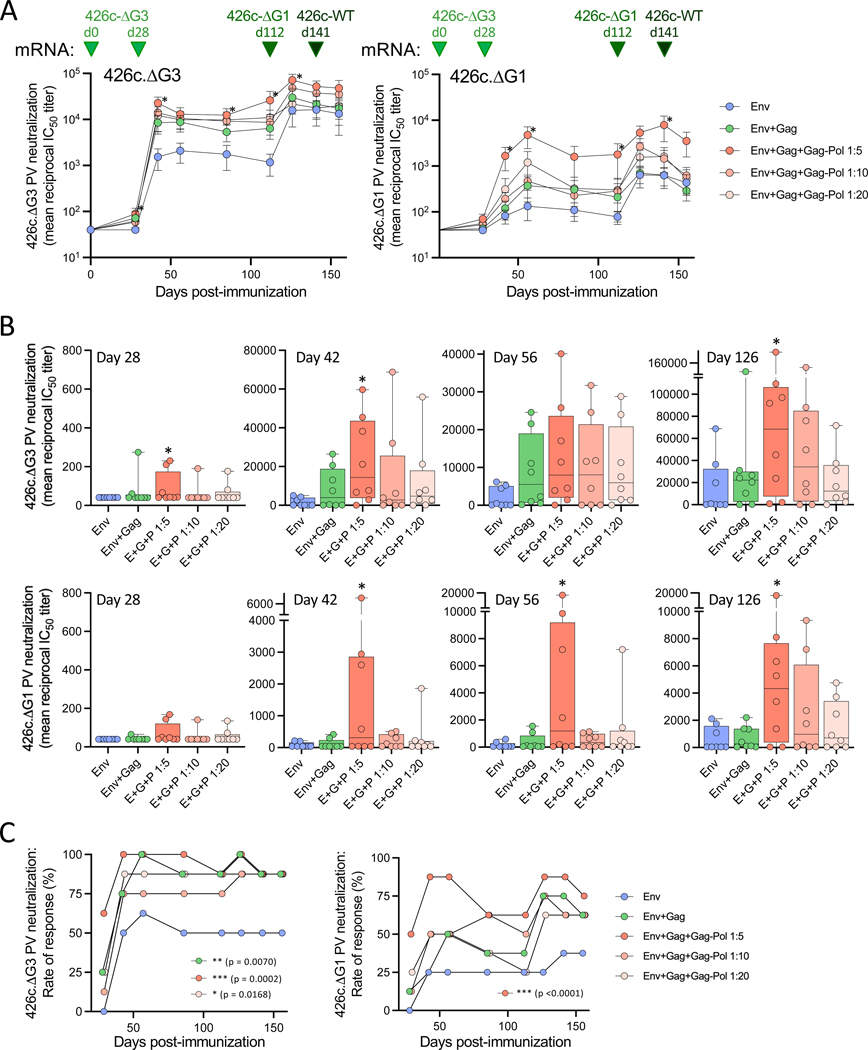
Neutralizing antibody titers in serum of mice immunized with mRNA producing immature or mature HIV-1 VLPs. (**A**) Neutralization of autologous 426c-ΔG3 (left panel) and 426c-ΔG1 (right panel) Env pseudoviruses (PVs) over time in different groups of immunized mice. Data are presented as mean values (±SEM) of IC_50_ titers for each study arm. (**B**) Neutralization of HIV-1 426c.ΔG3 (upper row) and 426c.ΔG1 (lower row) PVs by sera from individual immunized mice at day 28, 42, 56, and 126. The data indicate IC_50_ values shown in box-and-whisker representation with individual points denoted by the circles, upper and lower quartiles by the box, and median values by the horizontal line; whiskers represent the minimum and maximum values. (**C**) Rate of serum neutralization response against HIV-1 426c.ΔG3 (left panel) and 426c.ΔG1 (right panel) PVs in different study arms. The data indicate the proportion of mice in each group with neutralization titers over background values at each time point. All statistical comparisons were made by non-parametric Kruskal-Wallis test followed by post-hoc Dunn’s correction for multiple comparisons with Arm 1. The asterisks indicate significant *p*-values: * < 0.05; ** < 0.01; *** <0.001.

Next, we tested the neutralizing activity of serum collected from immunized mice at different time points against pseudoviruses expressing the priming Env, 426c-ΔG3, and the first-boost Env, 426c-ΔG1. A wide variability was observed in the rate and magnitude of neutralizing antibody responses even within individual vaccine groups, likely as a consequence of the relative rarity of precursor B cells capable of recognizing HIV-1 neutralization epitopes in WT mice, as well as the inefficient recruitment of such precursors by the suboptimal mRNA doses utilized in this study. Occasional animals in all groups except Arm 1 started to show low titers of 426c-ΔG3-neutralizing antibodies after a single immunization (day 28) (Fig. 5, A and B). Two weeks after the second immunization (day 42), there was an increase in neutralization titers, which was more marked in the three Pro-containing Arms, especially in Arm 3 (1:5 *gag-pol*:*gag* ratio) in which 4 of the 8 animals developed half-maximal inhibitory concentration (IC_50_) titers greater than 1:20,000; Arm 3 was the only group with significantly higher neutralization titers than Arm 1 (p = 0.0175), as analyzed by Kruskal-Wallis test followed by Dunn’s correction for multiple comparisons (Fig. 5B). Neutralization titers in Arm 3 remained significantly higher at day 85 (p = 0.0375) and 112 (p = 0.0348), as well as after the third immunization (day 126) (p = 0.0406) when 5/8 mice (62.5%) in this group had IC_50_ titers greater than 1:40,000, compared to 4/8 (50%) in Arm 4, 1/8 (12.5%) in Arm 5 and none (0%) in Arms 1 and 2, which were not immunized with Pro-containing regimens (Fig. 5B).

Neutralization titers against 426c-ΔG1, which features a more protected CD4-BS, were overall lower than against 426c-ΔG3 at all time points, but Arm 3 showed again a consistently higher response than the other groups, with significantly higher titers than in Arm 1 on days 42, 56, 126 and 141 (all p < 0.05) (Fig. 5, A and B). On day 126, 5/8 mice (62.5%) in Arm 3 had neutralization titers greater than 1:3,000 against 426c-ΔG1, compared to 2/8 (25%) in both Arms 4 and 5, and none (0%) in Arms 1 and 2 (Fig. 5B). The increased immunogenicity of the Pro-containing vaccine regimens was confirmed by analysis of the mean AUCs for neutralization titers against both 426c-ΔG3 and 426c-ΔG1 (table S1). Moreover, comparison of the rates of neutralization response over the entire course of the study showed that Arm 3 had the highest response rates against both 426c-ΔG3 and 426c-ΔG1 (both p < 0.001; Fig. 5C). Altogether, the trimer-binding and neutralization results demonstrate that the addition of Pro in the form of *gag-pol* mRNA increases the immunogenicity of a VLP-forming HIV-1 *env-gag* mRNA vaccine and identify a 1:5 Wt:Wt ratio between *gag-pol* and *gag* mRNA as the most immunogenic in a pre-clinical mouse model.

### Addition of SIV Pro induces the production of mature Gag-based SARS-CoV-2 VLPs

**Fig. 6.**
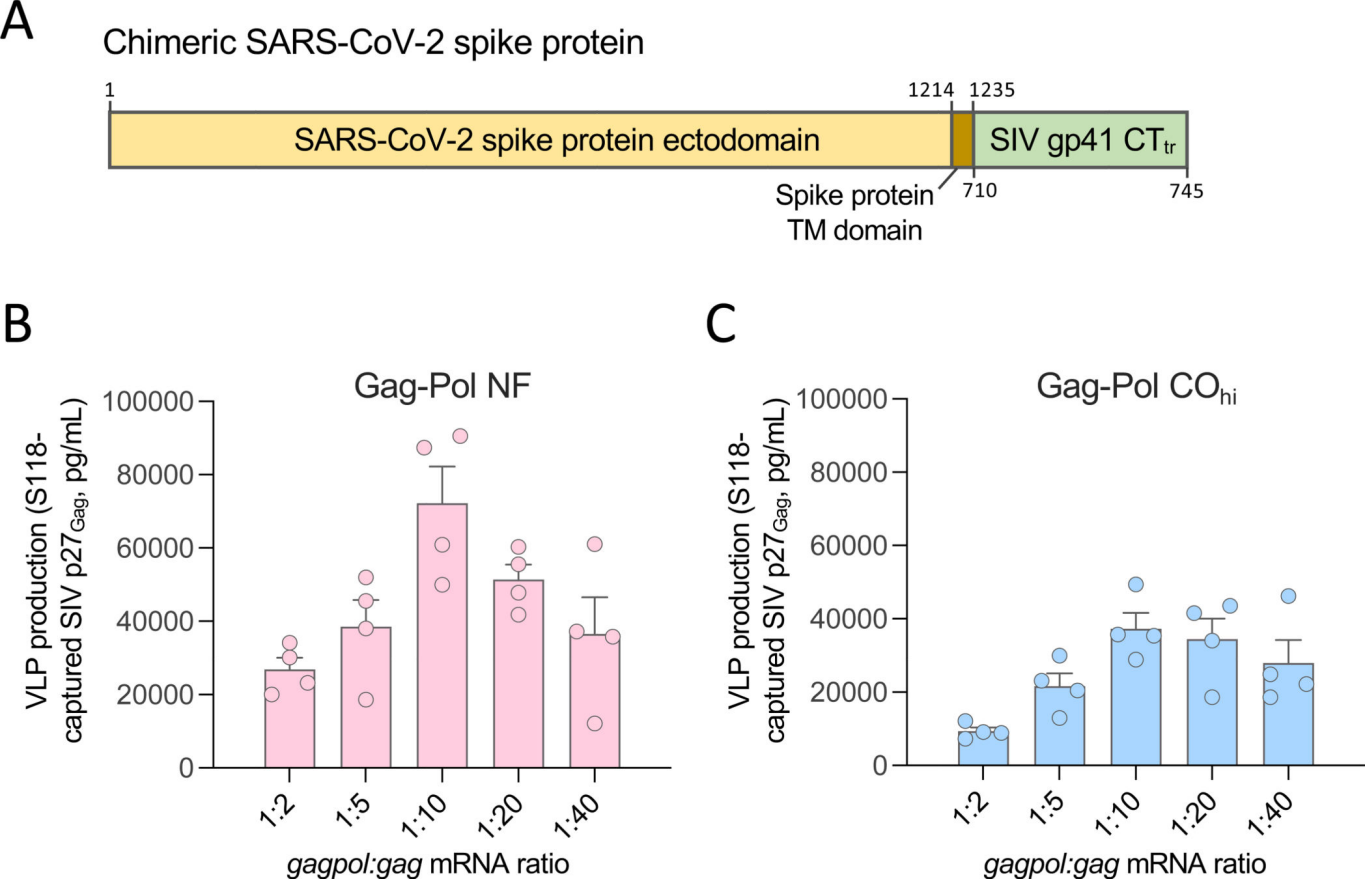
Design of a chimeric SARS-CoV-2 vaccine platform forming mature VLPs. (**A**) Schematic representation of the chimeric SARS-CoV-2 spike protein (Spike-S) utilized in this study, encompassing the SIV gp41 cytoplasmic tail, truncated at aa. 745, fused to the C-terminus of the transmembrane domain of the Wuhan-1 spike protein. (**B**) Quantification of extracellular VLPs produced by HEK293T cells co-transfected with mRNA encoding the chimeric Spike-S protein (1 μg per reaction), SIV *gag* (1 μg per reaction), and decreasing amounts of Gag-Pol NF (at the ratios indicated on the *x* axis) collected at 48 hours post-transfection. (**C**) Quantification of extracellular VLPs produced by HEK293T cells co-transfected with mRNA encoding the chimeric Spike-S protein (1 μg per reaction), SIV *gag* (1 μg per reaction), and decreasing amounts of Gag-Pol CO_hi_ (at the ratios indicated on the *x* axis) collected at 48 hours post-transfection. VLPs were captured from the culture supernatants 48 hours after transfection using the spike protein-specific monoclonal antibody S118 bound to magnetic beads. The amount of VLP-associated Gag p27 was quantified using an ELISA specific for cleaved SIV p27. Data are presented as the mean (± standard error of the mean) from two technical replicates from a representative experiment out of three performed.

To evaluate the applicability of our mature VLP-mRNA vaccine platform to other infectious agents besides HIV-1, we utilized a hybrid VLP platform that we recently reported to improve the immunogenicity of a SARS-CoV-2 mRNA vaccine (40). First, we tested the efficiency of mature VLP production using a chimeric SARS-CoV-2 spike protein based on the sequence of the original Wuhan-1 strain, fused with the cytoplasmic tail of SIV gp41, truncated at aa. 745 (Spike-S, Fig. 6A). Although expressed less efficiently in transfected HEK-293T cells than the native spike protein, this chimeric spike protein was found to be the most efficient for the extracellular production of SIV Gag-based VLPs, presumably due to cognate interaction between SIV Gag and the cytoplasmic domain of SIV gp41 (40). Thus, mRNA encoding Spike-S was co-transfected with SIV *gag* mRNA in the presence or absence of two different SIV *gag-pol* mRNA constructs, SIV *gag-pol CO*_*hi*_ or *gag-pol NF,* at different Wt:Wt ratios. As seen with the HIV-1 Env, abundant extracellular VLP production was detected with both *gag-pol* mRNA constructs (Fig. 6, B and C). Based on these results, we concluded that SIV Pro can be incorporated into an SIV Gag-based, VLP-forming SARS-CoV-2 mRNA vaccine.

### Inclusion of SIV Pro enhances the immunogenicity of a VLP-forming SARS-CoV-2 mRNA vaccine in mice

**Fig. 7.**
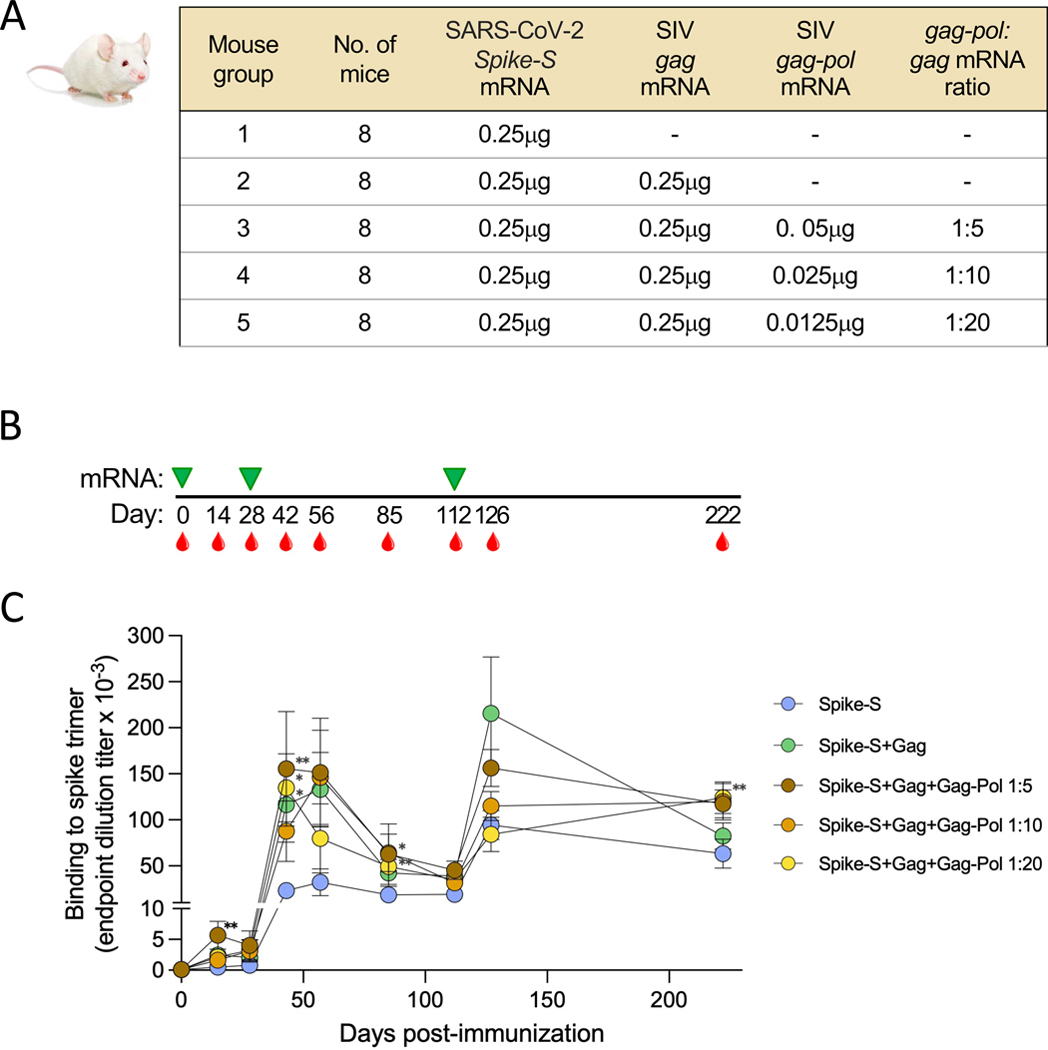
Immunogenicity of mRNA vaccines producing immature or mature SARS-CoV-2 VLPs in mice. (**A**) Design of the immunization study. Each vaccine arm included eight wild-type BALB/c mice. Mice were immunized with in LNP-formulated mRNA encoding chimeric SARS-CoV-2 Spike-S and SIV mac239 Gag with or without Gag-Pol NF. (**B**) Schematic time course of immunizations (green arrows) and bleedings (red drops). All the immunizations were performed with the same *Spike-S* mRNA with or without SIV *gag* and *gag-pol* mRNA. (**C**) Induction of SARS-CoV-2 spike trimer-binding antibodies in the 5 study arms over time, as assessed by ELISA. Data are presented as mean values (±SEM) of endpoint titers for each study arm. Statistical comparisons were made by Kruskal-Wallis test followed by post-hoc Dunn’s correction for multiple comparisons with Arm 1. The asterisks indicate significant *p*-values: * < 0.05; ** < 0.01.

Based on the in vitro results obtained with the chimeric SARS-CoV-2 Spike-S protein, we designed a pre-clinical study in mice to compare the immunogenicity of mRNA formulations expressing membrane-bound chimeric Spike-S either alone or in combination with SIV *gag* mRNA plus or minus different amounts of SIV Pro in the form of *gag-pol NF* mRNA. The study design was similar to that described above for anti-HIV-1 immunization. BALB/c mice (n=8 per Arm) were immunized with 5 different mRNA combinations (Fig. 7A). As in the HIV-1 immunization study, a sub-optimal amount of Spike-S-encoding mRNA was used (0.25 μg/dose). The mice were immunized three times, on days 0, 28 and 112, with the same immunogens (Fig. 7B). Spike protein-binding antibodies, as measured by end-point dilution immunoassays performed with plastic-immobilized S2P trimers (Wuhan-1 strain), started to be detectable at low titers in all groups after the first immunization, but increased dramatically after the second immunization (day 43 and 56) and again after the third immunization (day 126) following a decline on day 85. In agreement with previous observations (40), the VLP-forming mRNA formulation (Arm 2) induced higher titers than Spike-S-encoding mRNA alone (Arm 1) on day 42, as assessed by Kruskal-Wallis test followed by Dunn’s correction for multiple comparisons (p = 0.0168 Fig. 7C). In contrast, the regimens including Pro showed significantly higher titers at multiple time points: Arm 3 on days 14 (p = 0.0048) and 42 (p = 0.0062), Arm 4 on days 85 (p = 0.0363), and Arm 5 on days 42 (p = 0.0196), 85 (p = 0.0037) and 222 (p = 0.0044) (Fig. 4C). The mean AUCs for trimer-binding antibodies in each vaccine Arm showed a similar trend (table S2).

**Fig. 8.**
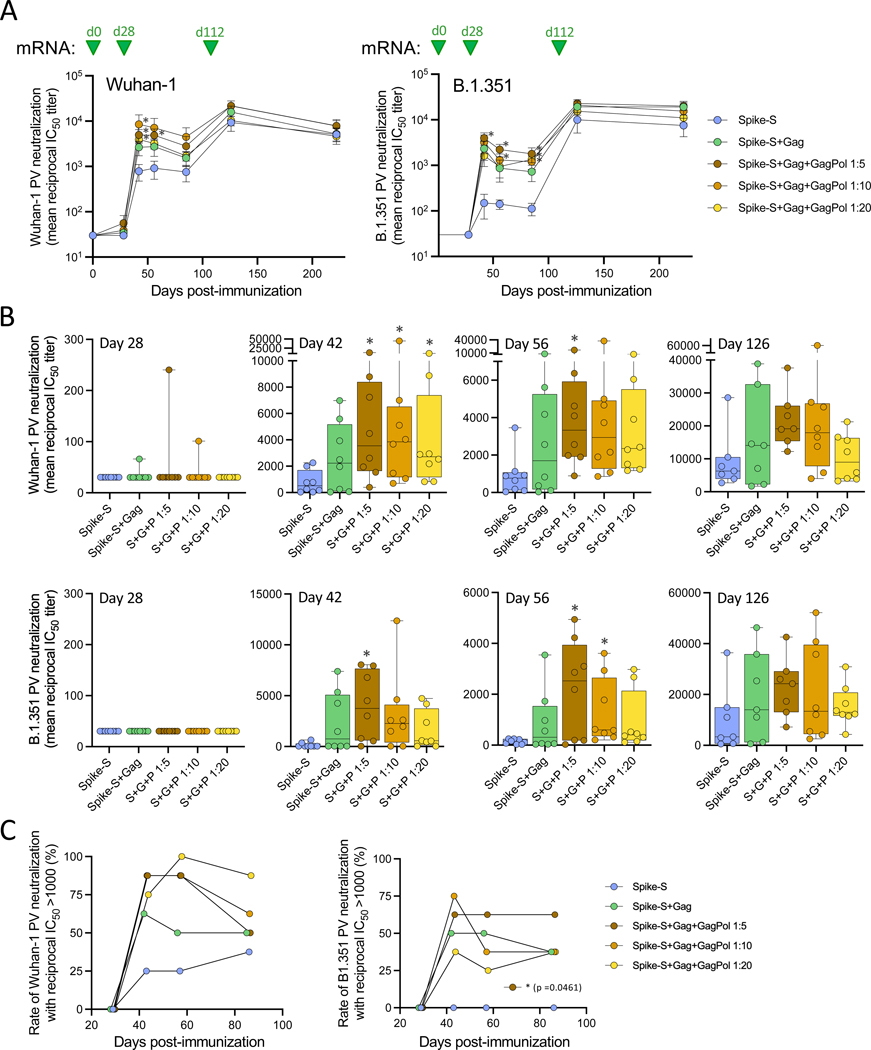
Neutralizing antibody titers in serum of mice immunized with mRNA producing immature or mature SARS-CoV-2 VLPs. (**A**) Neutralization of autologous (Wuhan-1, left panel) and heterologous (B.1.351, right panel) SARS-CoV-2 pseudoviruses (PVs) over time in immunized mice. The data are presented as mean values (±SEM) of half-maximal neutralization titers (IC_50_) for each study arm. (**B**) Neutralization of autologous (Wuhan-1, left panel) and heterologous (B.1.351, right panel) PVs by sera from individual immunized mice at day 28, 42, 56, and 126. The data indicate IC_50_ values shown in box-and-whisker representation with individual points denoted by the circles, upper and lower quartiles by the box and median values by the horizontal line; whiskers represent the minimum and maximum values. (**C**) Rate of serum neutralization responses with IC_50_ greater than 1:1000 against SARS-CoV-2 Wuhan-1 (left panel) and B.1.351 (right panel) PVs in different study arms at all time points before the third immunization. The data indicate the proportion of mice in each group with reciprocal neutralization titers over 1000 at days 28, 42, 56, and 85. All statistical comparisons were made by non-parametric Kruskal-Wallis test followed by post-hoc Dunn’s correction for multiple comparisons with Arm 1. The asterisks indicate significant *p*-values: * < 0.05; ** < 0.01; *** <0.001.

Next, we tested the pseudovirus neutralizing activity of the immune mouse sera collected at different time points after immunization. The first testing was performed against the autologous strain used for immunization (Wuhan-1). Only occasional animals showed neutralization titers after a single immunization (day 28); however, two weeks after the second immunization (day 42) there was a brisk rise in neutralization titers, with the three groups receiving Pro-supplemented vaccines (Arms 3, 4 and 5) showing significantly higher titers than Arm 1 by Kruskal-Wallis test followed by Dunn’s correction for multiple comparisons (p < 0.05) (Fig. 8, A and B). The same trend persisted for Arm 3 on day 56 (p = 0.0210), although neutralization titers became more homogeneous among the 5 Arms after the third immunization on day 112 (Fig. 8, A and B).

Then, we tested the neutralizing activity of the mouse sera against a pseudovirus bearing the spike protein of a heterologous SARS-CoV-2 strain, B.1.531. Neutralization was first detected after the second immunization, on day 42, when only Arm 3 showed significantly higher titers than Arm 1 (p = 0.0105) (Fig. 8, A and B). All the groups immunized with VLP-forming mRNA regimens had neutralization titers of comparable magnitude with those against the autologous Wuhan-1 strain, unlike Arm 1 that showed markedly lower titers (Fig. 8, A and B). For example, the mean IC_50_ values in Arm 3 were 4,969±4,284 against Wuhan-1 and 3,956±3,361 against B.1.351. These results corroborate the concept that VLP-forming immunogens are efficient at eliciting broad neutralization. Arms 3 and 4 had significantly higher neutralization titers than Arm 1 on both days 56 and 85 (p < 0.05), whereas Arm 5 neutralization titers were significantly higher than Arm 1 only on day 85 (p = 0.0325) (Fig. 8, A and B). In line with the dramatic boost in neutralization titers seen in humans after the second COVID-19 vaccine boost, Arm 1 showed the most pronounced increase in titers following the third immunization on day 112, which markedly reduced the gap with the other groups (Fig. 8A). When the rates of neutralization response with reciprocal titers greater than 1000 before the third immunization were compared, only Arm 3 had a significantly higher rate than Arm 1 against the heterologous strain, B.1.531 (p = 0.0461) (Fig. 8C). The increased immunogenicity of the Pro-containing vaccine regimens was confirmed by analysis of the mean AUCs for neutralization titers against both Wuhan-1 and B.1.531 (table S2). In summary, the above results confirmed in a hybrid vaccine platform against a second, unrelated viral agent, SARS-CoV-2, that the addition of SIV Pro can increase the immunogenicity of VLP-forming mRNA vaccines.

## DISCUSSION

The advent of mRNA has ignited remarkable progress in the field of vaccinology, especially owing to the extraordinary versatility and rapidity of development of this platform (50–52). In the HIV-1 vaccine area, mRNA offers some key advantages over protein-based immunogens because it directs the endogenous manufacturing of the viral Env glycoproteins in a *bona fide* native form by the host cells. Endogenous expression implies that Env will be decorated with native N-linked glycosylation, which is essential for several conserved neutralization epitopes targeted by bNAbs to adopt their correct antigenic conformation (53–55). This is a critical advantage of mRNA and vector-expressed vaccines for targeting highly glycosylated proteins such as the HIV-1 Env. We have taken advantage of the great versatility of the mRNA technology to devise a vaccine platform suited for the production of VLPs, which are believed to be more effective immunogens compared with conventional subunit vaccines (56, 57). The main advantages of VLPs derive from the fact that they closely mimic the native viral particles produced during natural infection in terms of size, shape and antigenicity. Size matters in immunology because particulate antigens, especially those within a specific size range, are more efficiently recognized and taken up by antigen-presenting cells (38, 58, 59). Likewise, shape matters too, as spheric particles were found to be more palatable for antigen-presenting cells than other shapes (60). But the property that arguably can make VLPs even more effective as immunogens is the presentation of native antigens in repetitive arrays on their surface, which provides the most effective mode for B cell receptor (BCRs) cross-linking and, as a consequence, B cell activation (61). Our VLP-forming, HIV-1 *env-gag* mRNA vaccine platform was tested in both mouse and macaque models and found to be highly effective in inducing HIV-1 trimer-binding and neutralizing antibodies; furthermore, in macaques, it induced protection from mucosal virus challenges with a difficult-to-neutralize heterologous tier-2 SHIV (39). More recently, we adapted a similar Gag-based VLP/mRNA platform to SARS-CoV-2 and documented its immunogenicity in a mouse model (40). In the present report, we introduce a key technological advance to improve the original VLP-forming mRNA platform with the inclusion of a retroviral protease, the specific enzyme that processes the Gag polyprotein precursor (p55) to generate its cleavage products, thereby leading to the production of mature, rather than immature, VLPs.

A major technical hurdle that we faced in the first phase of the study was the design of an effective construct to express a retroviral protease by mRNA. Retroviral proteases are potentially toxic enzymes, with a relatively narrow window between the minimal effective and maximal non-toxic concentrations, and require a precisely timed activation in order to avoid premature cleavage of the viral capsid before p55 becomes anchored to the plasma membrane through its myristoylated N-terminal domain (62, 63). If the protease is activated too early, the cleaved capsid protein (p24 for HIV-1, p27 for SIV) is released free into the cytosol, with only a minor fraction reaching the site of virion budding for incorporation into nascent viral particles. Indeed, our initial experiments with an mRNA construct encoding only the protease gene were not successful, yielding only a very limited production of Gag-containing extracellular VLPs. To overcome this drawback, we decided to take inspiration from the natural process adopted by HIV-1 and SIV, which transcribe a long Gag-Pol precursor mRNA that enables high expression of Gag but lower expression of the three viral enzymes (protease, reverse transcriptase, and integrase), which is required for them to carry out their catalytic functions. Thus, we the synthesized and validated multiple SIV Gag-Pol constructs with or without the ribosomal slippery site that mediates the reading frameshift for the translation of the enzymes at the ribosomal level. We selected the SIV Gag-Pol, instead of HIV-1, because our aim was to design a vaccine suitable for testing in a challenge model in rhesus macaques, which are susceptible to SHIV infection and pathogenesis (64). Measurement of extracellular release of mature VLPs containing cleaved capsid proteins was used to guide our choice for use in our mRNA vaccines. The most effective Gag-Pol construct was one lacking the ribosomal frameshift mechanism, with all the genes on the same reading frame and fully codon optimized. To appropriately dose the amount of protease expression, we tested different ratios between Gag- and Pro-encoding mRNAs. The results consistently showed optimal VLP production in the range between 1:5 and 1:20 *gag-pol*:*gag* mRNA ratios. The particles were visualized by EM and showed a relatively homogeneous size and shape with a mean diameter of 100 μm and a rich complement of Env spikes that appeared to occupy the entire VLP surface.

Having defined the optimal in vitro conditions for the production of mature VLPs, we aimed at comparing the in vivo immunogenicity of the triple mRNA formulation (*env+gag+gag-pol*) versus our original *env+gag* mRNA formulation. To evaluate the vaccines under physiological conditions, we utilized a non-transgenic mouse model and selected an HIV-1 Env, 426c-ΔG3, which displays an open CD4-BS due to the ablation of three surrounding N-linked glycans, N276, N460 and N463, and thus could be more easily recognized by the native murine antibody repertoire. To highlight the potential added value of the protease inclusion, we utilized a suboptimal dose of *env* mRNA (only 2.5 μg per immunization). As expected, due to such low immunogen dose, we observed highly variable immune responses in our immunized mice. Nevertheless, we observed a consistent increase in the titers of both Env trimer-binding and neutralizing antibodies in mice immunized with the Pro-containing regimens, which generate mature VLPs, compared with mice immunized with *env* mRNA alone or with the original *env+gag* mRNA formulation, which generates only immature VLPs. In addition, mice immunized with the Pro-containing formulation showed higher rates of response. Importantly, NAb titers in mice immunized with the Pro-containing formulation at 1:5 *gag-pol*:*gag* ratio (Arm 3) were generally superior to those measured in the other groups, including against a more challenging Env form, 426c-ΔG1, which is deglycosylated only at a single site (aa. 276) and, therefore, harder to recognize by vaccine-elicited antibodies.

Next, we tested the potential benefits of retroviral protease inclusion in a VLP-forming vaccine against a different virus, SARS-CoV-2. We previously reported the adaptation of our retroviral Gag-based mRNA platform to a SARS-CoV-2 vaccine through engineering of the spike protein to foster homologous recognition with SIV Gag and, thereby, promote VLP formation (40). Thus, we used a modified SARS-CoV-2 spike protein where the C-terminus of the native transmembrane domain was fused with a partially-truncated cytoplasmic tail of SIV gp41. Co-expression of the resulting hybrid protein with SIV Gag and Gag-Pol resulted in the efficient production of mature VLPs in vitro. The immunogenicity and effectiveness of this vaccine were assessed in mice using a protocol analogous to that used for HIV-1. Again, we used only a low dose of spike protein-encoding mRNA, which resulted in variability in antibody responses within each group. Although the titers measured after the first immunization were very low, as expected due to the suboptimal amount of mRNA utilized, they showed a substantial rise after the second and especially after the third immunization. Overall, both trimer-binding and neutralizing antibody responses were clearly and consistently higher in the Pro-containing vaccine groups than in the other groups. As seen against HIV-1, the differences were more pronounced against the most challenging virus, the heterologous strain, B.1.351. However, the differences among groups were considerably reduced after the third immunization, when the group immunized with the spike protein-encoding mRNA alone showed the most dramatic rise in neutralization titers against both the homologous and heterologous virus, in agreement with data reported for human SARS-CoV-2 mRNA vaccines (65, 66). Together, the results obtained with the hybrid Gag-based SARS-CoV-2 vaccine confirmed the improvements achieved with the inclusion of the protease, especially during the early phase of vaccination.

The mechanism underlying the superior immunogenicity of vaccines producing mature versus immature VLPs is unclear. In immature HIV-1 viral particles, the Env spikes have a restricted lateral movement due to interaction of the Env C-terminus with the underlying rigid Gag lattice, which prevents the formation of Env clusters; in contrast, mature viral particles were shown to possess a greater mobility and to form Env clusters on the virion surface, which are predicted to be more effective triggers of B cell receptor cross-linking and activation (42, 67, 68). Another previous study demonstrated that mice immunized with pre-formed mature HIV-1 VLPs developed a broader range of specific antibody isotypes than mice immunized with immature VLPs (33). Further studies on the ultrastructural and functional features of the different VLP forms will be instrumental to elucidate the mechanistic basis for the improvement brought upon by Gag processing and presentation of the viral spike in the context of mature, rather than immature VLPs.

A limitation of the current study was the lack of pre-clinical evaluation of our Pro-containing vaccines in animal models suitable for protection studies, such as macaques challenged with SHIV. Although the data obtained in our mouse models indicate a clear benefit of Pro inclusion in terms of elicitation of both binding and neutralizing antibody responses, it is difficult to predict if such enhanced responses would translate into improved vaccine-elicited protection. It has to be emphasized that, at least for HIV-1, the induction of protective immune responses by a vaccine remains a challenging goal. The case is different for SARS-CoV-2, for which both Syrian Golden hamsters and macaques can be protected from live virus challenge through vaccination. Challenge studies in relevant pre-clinical models will therefore be critical to confirm the improved effectiveness of Pro-containing VLP-forming mRNA vaccine platforms in eliciting protective immunity. Another limitation of this study was the use of pseudovirus-based assays to assess neutralization, which are performed using engineered cell lines and clonal viral glycoproteins. The limited availability of serum from mice at each time point precluded the confirmation of neutralization titers in more physiological assay systems.

In summary, the present study provides a means to improve the immunogenicity of Gag-based VLP-forming vaccine platforms by introducing a retroviral protease for the production of mature VLPs. In addition to confirming the superior immunogenicity of particulate immunogens over protein subunits, our results demonstrate that not only the size, but also the quality of the immunogen may have a major impact on the magnitude and effectiveness of immune responses.

## MATERIALS AND METHODS

### Study design

The main objectives of our study were to develop a suitable mRNA construct for the correctly timed and dosed expression of a retroviral protease (Pro) and to evaluate the immunogenicity of Pro-containing triple mRNA formulations (*env/spike+gag+gag-pol*), which produce mature VLPs, in pre-clinical mouse models. Thus, immunization with *env* mRNA alone (no VLP production) or *env+gag* mRNA (immature VLP production) was compared with triple mRNA formulations (*env/spike+gag+gag-pol*) in two different vaccine models, one against HIV-1 and one against SARS-CoV-2. To highlight potential differences between Pro-containing and non-Pro-containing vaccine groups, sub-optimal amounts of HIV-1 Env-encoding mRNA (2.5 μg/dose) or SARS-CoV-2 spike protein-encoding mRNA (0.25 μg/dose) were used for immunization. To determine the most effective *gag-pol* mRNA dose in vivo, three different Pro-containing formulations were evaluated in parallel, at 1:5, 1:10 or 1:20 *gag-pol:gag* mRNA ratios. The number of mice included in each immunization group (n=8) was judged to be sufficient to detect clear differences between groups. Mice were randomly assigned to groups; no blinding was used. Vaccine effectiveness was assessed by measuring serum titers of trimer-binding and neutralizing antibodies at various time points.

### mRNA immunogens

Sequence-optimized mRNAs encoding the immunogens were synthesized in vitro using an optimized T7 RNA polymerase-mediated transcription reaction with N1-methylpseudouridine-5’-triphosphate utilized in place of uridine-5’-triphosphate. The in vitro transcription DNA template contained the target open reading frame flanked by 5’ and 3’ untranslated regions and an encoded polyA tail. After transcription, a cap 1 structure was added enzymatically using vaccine capping enzyme and 2’O-methyltransferase (New England Biolabs, Cat. no. M0366L). Purified mRNA was sterilized by filtration and co-formulated into the same lipid nanoparticle (LNP) as previously reported (39). Briefly, mRNA was diluted in acetate, pH 5.0, and mixed with lipids (SM-102:DSPC:Cholesterol:PEG2000-DMG) dissolved in neat ethanol at a ratio of 3:1 (mRNA:lipids). The product was then dialyzed against phosphate-buffered saline (PBS), pH 7.4, tested for encapsulation, particle size/polydispersity, mRNA purity, and endotoxin, and was deemed acceptable for in vivo use.

### Animals and immunization protocol

BALB/c mice (6 to 8 weeks old) were obtained from Charles River. The experiments in mice were carried out in compliance with all pertinent US National Institutes of Health (NIH) regulations and were approved by the Animal Care and Use Committee (ACUC) of Moderna. Each mouse was injected with mRNA encoding HIV-1 Env (426c-ΔG3, 426c-ΔG1 or 426c-WT) or SARS-CoV-2 chimeric spike protein (Spike-S) (strain Wuhan-1) with or without mRNA encoding SIV Gag and SIV Gag-Pol NF. The different mRNAs were pre-mixed in sterile saline solution and inoculated in a volume of 0.05 mL using a sterile syringe by intramuscular injection in the hind leg. The HIV-1 vaccine was administered at weeks 0, 4, 16 and 20; the SARS-CoV-2 vaccine at weeks 0, 4 and 16. Blood was obtained by retro-orbital collection on day 0, 14, 28, 42, 56, 85, 112, 126 and 155; for the SARS-CoV-2 vaccine, an additional bleeding was performed at day 222. Blood collections did not exceed 1% of the total body weight or 10 μL/g over a 2-week period.

### Protein expression and production

HIV-1 SOSIP.664 trimers and SARS-CoV-2 spike protein soluble trimers were expressed by co-transfecting human embryonic kidney (HEK)-293 free-style (FS) cells with plasmids expressing the respective trimer and the cellular protease furin. Cell-free supernatants were harvested after 7 days, passed through a 0.22 μm filter, and loaded onto a *Galanthus nivalis* (GNA) lectin column (Vector Laboratories, Cat. no. AL-1243–5). After washing with PBS, trimer proteins were eluted with 0.3 M methyl α-D-mannopyranoside in PBS, followed by size exclusion chromatography using a Superdex 200 16/600 PG column (Cytiva, Cat. no. 28989335). The purified trimers were concentrated to 1 to 2 ml using Amicon Ultra-50 centrifugal filter units (MWCO 50,000, Millipore Sigma, Cat. no. UFC9050) and stored at −80°C.

### Flow cytometry

To evaluate the cellular surface expression of HIV-1 Env and SARS-CoV-2 spike protein, the corresponding plasmids were transiently transfected into HEK-293T/17 cells (ATCC, Cat. no. CRL-11268) as described (69). After 48 hours, cells were harvested by mechanical shaking and pipetting, washed with FACS buffer (PBS supplemented with 2% FBS) and incubated with anti-gp120 human monoclonal antibodies (0.5 μg/ml; obtained through the AIDS Reagent Program) or anti-SARS-CoV-2 spike protein S-118 (1 μg/ml; a gift from the Vaccine Research Center, NIH) for 30 min at 4°C. After washing with FACS buffer, the cells were incubated with phycoerythrin (PE)-conjugated sheep anti-human IgG at 1:500 dilution (eBioscience, Cat. no. PA1–28652) for 30 min at 4°C. The cells were then washed twice with FACS buffer, fixed in 2% paraformaldehyde in PBS, and analyzed on a BD Fortessa (BD Biosciences). Data analysis was performed using the FlowJo software (V9).

### In vitro transfection, VLP capture, and VLP quantification

mRNAs encoding different HIV-1 Env, SIVmac239 Gag, SIV Pro or different Gag-Pol constructs were co-transfected into HEK-293T cells. The relevant mRNAs were pre-mixed and added to 6-well plates (10^6^ cells per well) together with 6 μL of transfection reagent and transfection booster from TransIT-mRNA Transfection Kit (Mirus Bio, Cat. no. MIR 2225). Supernatants were harvested 48 hours after transfection, centrifuged at 1,000 rpm and filtered through a 0.45 μM PES filter (Millipore Sigma, Cat. no. HPWP04700). Cell viability was assessed using a cell viability kit (Cytek, Cat. no. 4000–0340) on a Muse cell analyzer (Cytek). Total p27 Gag protein concentration in culture supernatants was measured using an in-house developed SIV p27 antigen ELISA. Serial dilutions of reference p27 protein and culture supernatants were coated onto 96-well ELISA plates (Corning, Cat. no. 9018) at 4ºC overnight. Then, the plates were blocked with 1x casein at room temperature for 1 hour, followed by the addition of mouse anti-p27 monoclonal antibody (AIDS Reagent Program, Cat. no. 3537) at 1 μg/ml. After washing, horseradish peroxidase (HRP)-conjugated goat anti-mouse IgG (Invitrogen, Cat. no. A16072) was added for 1 hour at room temperature. The optical density (OD) at 450 nm was measured using an ELISA plate reader (Biotek). The p27 concentration were calculated by dose-response curve fit with a 5-parameter nonlinear function using GraphPad Prism v8. For HIV-1 VLP capture from culture supernatants, the anti-Env bNAb PG16 was incubated with Protein G-conjugated immunomagnetic beads (Thermo Fisher, Cat. no. 10004D) at 20 μg/ml; for SARS-CoV-2 VLP capture, the anti-SARS-CoV-2 spike protein S-118 was incubated with the beads at 10 μg/ml. Antibody-armed beads were incubated with VLP-containing supernatants at room temperature for 30 minutes and then washed 3 times with PBS containing 0.025% (vol/vol) casein (Vector Laboratories, Cat. no. SP-5020–250) to remove the unbound material. Captured VLPs were lysed with RIPA buffer on the beads, and the eluted p27 Gag protein concentration was measured using our in-house p27 ELISA. For concentrating VLPs, 25 mL of VLP-containing supernatants were layered onto a 10 to 20% linear gradient sucrose PBS buffer and centrifuged at 29,300 rpm (Beckman Rotor Type 50.2 Ti, ~78,000 g) for 2 hours at 4ºC. After centrifugation, the supernatants were discarded and the VLP pellets were resuspended in PBS and stored at 4ºC or at −80ºC.

### Immunoblot analysis

To study the expression of SIV p55, we used immunoblot analysis with a mouse monoclonal antibody to SIV-p27 (AIDA Reagent Program, Cat. no. 3537). 293T cells transfected with SIV *gag* and *gag-pol* mRNA were lysed with RIPA buffer for 30 min on ice. After centrifugation, the cleared supernatant was treated with loading buffer, and the samples were resolved by SDS electrophoresis and immunoblotted. After blocking, the primary antibody was used to incubate at 1 μg/ml for overnight at 4°C. After washing, goat-anti-mouse HRP-conjugated antibody at 1:200 dilution (Invitrogen, Cat. no. A16072) was applied to the membrane for 1 hour at room temperature. The chemiluminescence signal was developed by ECL WB detection reagent (Cytiva, Cat. no. RPN2106) and the image was acquired by ProteinSimple imager (BioTechne).

### Cryo-electron tomography (cryo-ET) and image reconstruction

To make grids for cryo-ET, Quantifoil R 2/2, 300-mesh carbon grids (Electron Microscopy Sciences, Cat. no. Q3100CR2) were glow discharged for 30s. A 3.5 μl aliquot of VLP sample was applied to the grid and was allowed to adsorb on the grid for 10s inside a humidity-controlled chamber to avoid evaporation. The grids were then immediately plunge frozen in liquid ethane using a Vitrobot Mark IV (FEI Co.) at 4°C and 100% humidity with 3.5s blotting. Frozen grids were imaged using a 300 kV Krios with a Gatan K3 direct electron detector. Tilt-series were collected in a dose-symmetric tilting scheme from −60° to +60° or from with a step size of 3° using SerialEM. 70. Tilt-series were collected in Article counting mode at a magnification of 42000X, corresponding to a pixel size of 2.064 Å per pixel. The total dose per tilt series was ~100 e-/Å2. Tilt-series image frames were corrected for electron beam induced motion using Motioncor2. Tilt images were then batch processed using IMOD and Aretomo to generate 3-dimensional tomogram reconstructions. The final tomograms were binned, low pass-filtered and contrast enhanced in ImageJ for visualization and analysis. The density of Env spikes on VLP surfaces was counted manually.

### Trimer-binding assays

To assess the trimer-binding titers in the serum of immunized mice, 50 μl of Galanthus nivalis lectin (GNA) lectin (Millipore Sigma, Cat. no. L8275) at 1 μg/ml in PBS, pH 7.4, were coated onto 96-well plates at 4°C overnight. After incubation with blocking buffer (1x casein buffer in PBS) for 2 hours at room temperature, soluble HIV-1 426c-WT SOSIP trimers (containing the gp41 moiety of strain BG505) or SARS-CoV-2 Wuhan-1 spike trimers (both at 1 μg/ml) resuspended in binding buffer (0.1x casein in PBS) were added and incubated for 1 hour at 37°C. After repeated washing with washing buffer (R&D Systems, Cat. no. WA126), serially diluted mouse sera (5-fold dilutions, 7 steps from a starting dilution of 1:100) were incubated for 2 hours at room temperature, followed by incubation with alkaline phosphatase AffiniPure goat anti-mouse IgG (1:5000) for 1 hour at room temperature. The plates were then washed, and the reaction was developed by adding substrate reagents (R&D Systems, Cat. no. DY999). The reaction was stopped after 15 min by addition of sulfuric acid. Light absorbance at 450 nm was read by a plate reader (Enspire, Perkin Elmer) within 30 minutes. Endpoint binding titers were calculated by linear regression using Prism v10 after subtraction of background OD values obtained by the average of six control wells (no serum) plus twice the standard deviation from the same wells.

### Pseudovirus neutralization assays

HIV-1 pseudoviruses expressing WT or mutated gp160 from HIV-1 426c and derivative mutants (N276D/N460D/N463D or N276D) were produced in HEK 293T/17 cells (ATCC, Cat. no. CRL-11268) by co-transfecting Env-expressing plasmids with pSG3Δenv, a backbone plasmid (AIDS Reagent Program, Cat. no. 11051) as reported (39). To generate the pseudoviruses, 0.5 μg of each Env-expressing plasmid and 2 μg of the backbone plasmid were mixed in Opti-MEM medium (Gibco, Cat. no. 31985062); and 7.5 μl of LiFect293 Transfection Reagent (LifeSct, Cat. no. M0002) was added, followed by 15-min incubation at room temperature. The DNA-LiFect293 mixture was then added to the cells into a 6-well plate and incubated overnight at 37°C for 3 days. The supernatants containing pseudoviruses were harvested by centrifugation, followed by 0.45μM filter filtration and stored at −80°C before use. For infection, titrated pseudoviruses were mixed with 3-fold diluted heated inactivated mouse serum and incubated 30 min at room temperature. TZM-bl cells (AIDS Reagent Program, Cat. no. 8129) were added into 96-well flat-bottom plates at 10,000 cells/well in complete Dulbecco’s Modified Eagle Medium (DMEM) supplemented with 10% fetal bovine serum, 1% penicillin-streptomycin, and 3 μg/mL puromycin. The final volume of each well was 100 μl. Luciferase reporter gene was detected 2 days later after removing 50 μl of supernatant and adding 40 μl per well of Bright- Glo Luciferase Assay substrate (Promega, Cat. no. E2610). The lysates were transferred into 96-well flat white plates and the Relative Light Units (RLU) were measured with a luminometer (Perkin Elmer). All the samples were tested in duplicate wells. The SARS-CoV-2 pseudovirus neutralization assays were performed as previously described (70). Single-round pseudoviruses were generated by co-transfection of plasmids encoding the SARS-CoV-2 spike protein (Wuhan-1 or B.1.351), luciferase (pHR CMV Luc), a lentivirus backbone (pCMV ΔR8.2), and human transmembrane protease serine 2 (TMPRSS2) at a ratio of 1:20:20:0.3 into HEK293T/17 cells using the transfection reagent LiFect293 (LifeSct, Cat. no. M0002). The pseudoviruses were harvested after 72 hours. The supernatants were centrifuged, filtered through a 0.45 μM filter, aliquoted and titrated before the neutralization assay. Heat-inactivated mouse sera were clarified by rapid high-speed centrifugation and pre-diluted (3-fold dilutions) in complete DMEM. Each serum dilution (25 μL) was mixed with 25 μL of diluted pseudovirus in 96-well plates and incubated for 30 min at room temperature. ACE-2-expressing 293T cells (293T-hACE2.MF, a gift from Dr. Michael Farzan) were added into 96-well flat bottom plates at 10,000 cells/well in the cell culture medium in a final volume of 100 μL then cultured at 37°C with 5% CO2. Seventy-two hours later, the supernatant was carefully removing, the cells were lysed with 40 μL Bright- Glo Luciferase Assay substrate (Promega, Cat. no. E2610), and the luciferase activity was measured as described above.

### Statistical analyses

Individual-level data are presented in data file S1. Statistical differences between two groups (fig. S2) were calculated by unpaired two-tailed t-test; differences between five groups of vaccinated mice (Fig. 4, 5, 7 and 8) were calculated by Kruskal-Wallis test followed by Dunn’s post-hoc correction for multiple comparisons and shown as: no significant difference (no asterisk), *p* > 0.05; *p < 0.05; **p < 0.01; and ***p < 0.001. Calculation of half-maximal inhibitory concentrations (IC_50_) was performed using the log (agonist) versus normalized response (variable slope) nonlinear function in Prism v10 (GraphPad). All statistical analyses were performed using GraphPad Prism v10. All graphs were plotted using Prism v10.

## Supplementary Material

Supplementary Figures and Tables

Data file S1

MDAR checklist

## Data Availability

All data associated with this study are presented in the paper or supplementary materials. Plasmids or proteins are available from P.L. under a material transfer agreement with NIAID, NIH, to be requested to P.L. mRNA-LNP vaccine constructs can be made available from S.H. under a material transfer agreement with Moderna. The GenBank accession codes for the mRNA immunogens are: SIVmac239.gag, MZ362876; SIVmac239.gagpol WT, PV178701; SIVmac239.gagpol CO_low_, PV178697; SIVmac239.gagpol CO_hi_, PV178698; SIVmac239.gagpol NF, PV178699; SIVmac239.gagpol NF_tr_, PV178700; HIV-1.426c.ΔG3, PV178696; and HIV-1.426c.ΔG1, PV178695.
