# Supplementary material for: Inclusion of a retroviral protease enhances the immunogenicity of VLP-forming mRNA vaccines against HIV-1 or SARS-CoV-2 in mice
Source: Sci Transl Med. Author manuscript; Available in PMC 2025 Jun 10. (PMC12151460; doi:10.1126/scitranslmed.adt9576)
Supplement: Supplementary Figures and Tables [file NIHMS2079224-supplement-Supplementary_Figures_and_Tables.docx]

**Supplementary Materials for:**

**Inclusion of a retroviral protease enhances the immunogenicity of VLP-forming mRNA vaccines against HIV-1 or SARS-CoV-2**

by: Peng Zhang, Mamta Singh, Vada A. Becker, Jacob Croft, Yaroslav Tsybovsky, Vinay Gopan, Yuna Seo, Qingbo Liu, Denise Rogers, Huiyi Miao, Yin Lin, Daniel Rogan, Courtney Shields, Sayda M. Elbashir, Samantha Falcone, Isabella Renzi, Vladimir Preznyak, Elizabeth Narayanan, Guillaume Stewart-Jones, Sunny Himansu, Mark Connors, Kelly Lee, Andrea Carfi, and Paolo Lusso

**This PDF file contains:**

Figs. S1 to S3

Tbls. S1 and S2

**Other Supplementary Materials for this manuscript include the following:**

Data file S1

MDAR Reproducibility Checklist

**SUPPLEMENTARY FIGURES**

**
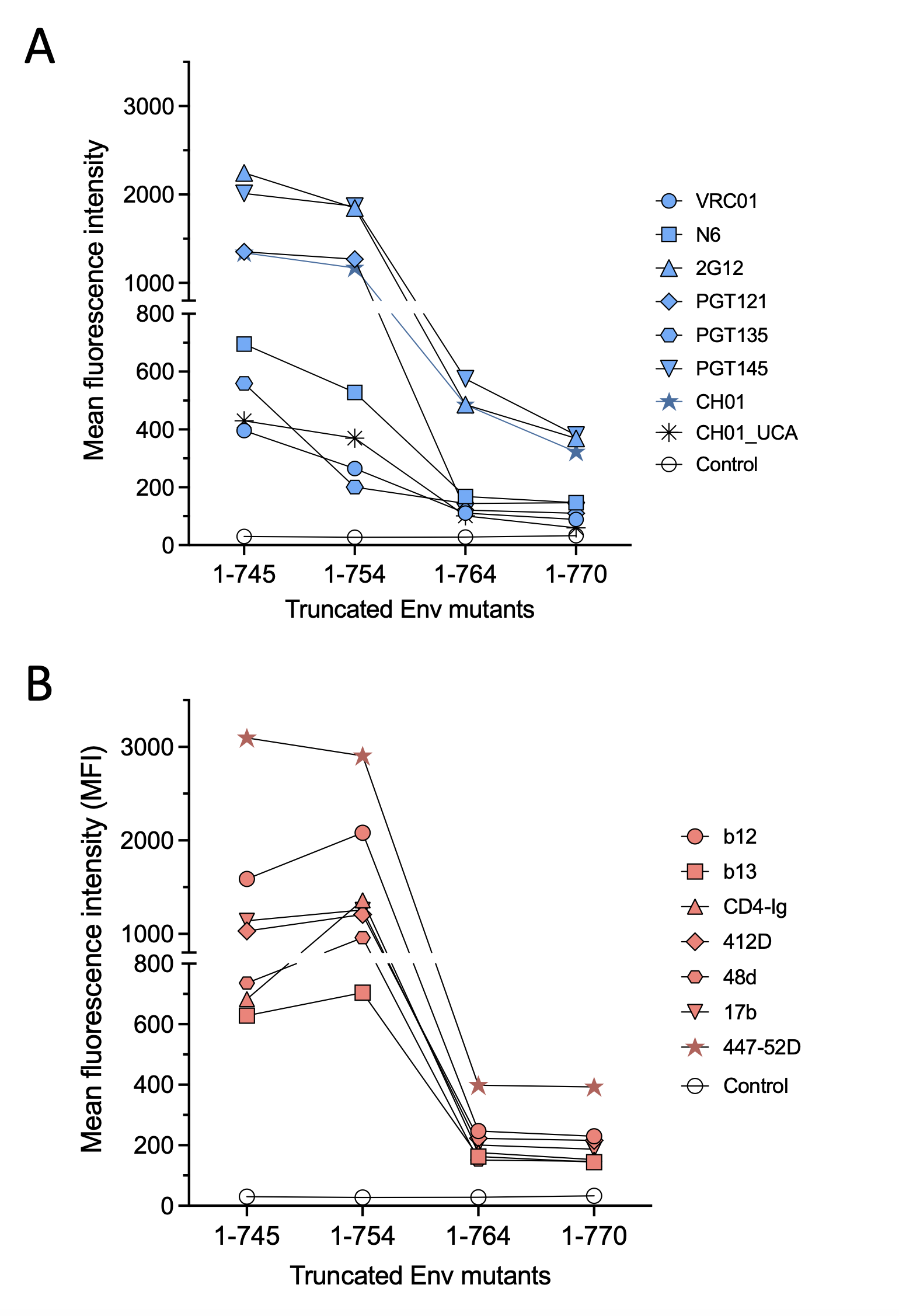
**

**Figure S1. Effect of gp41 C-terminal truncations on the expression and antigenic profile of the HIV-1 Env.** Flow cytometry analysis of transiently transfected 293T cells expressing different gp41 C-terminally (CT) truncated mutants, as indicated on the *x* axis. (**A**) Binding of broadly neutralizing antibodies (bNAbs) directed against different supersites of HIV-1 vulnerability, as well as the germline precursor of bNAb CH01 (CH01 UCA). (**B**) Binding of weakly/non-neutralizing antibodies (nNAbs) directed against different antigenic sites.

**
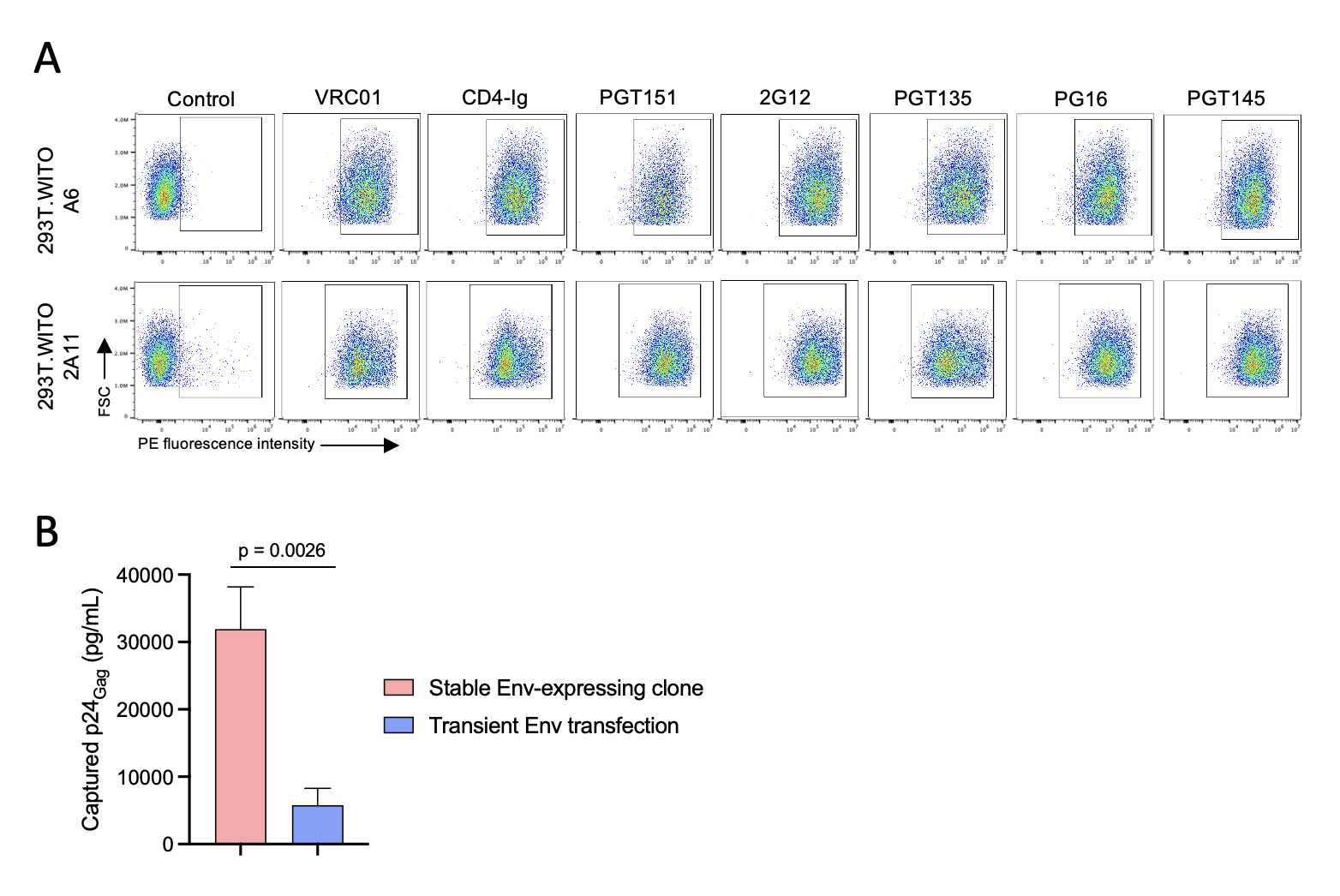
**

**Figure S2. Generation of stable Env-expressing clones for the production of HIV-1 VLPs.** (**A**) Flow cytometry analysis of the 293T-WITO.A6 and 293T-WITO.2A11 clone stably expressing the HIV-1 WITO.27_1.745 and WITO.27_G153E_1-745 Envs, respectively. The dot plots show binding of both conformation-independent bNAbs (e.g., 2G12) and conformation-dependent, trimer-specific bNAbs (e.g., PG16), as well as soluble CD4 (CD4-Ig). (**B**) Efficiency of extracellular VLP production by the stable Env-expressing clone 293T-WITO.2A11 vs. 293T cells transiently transfected with the WITO.27_ G153E_1.745 Env. The data represent the mean values (+SEM) from three independent experiments.

**
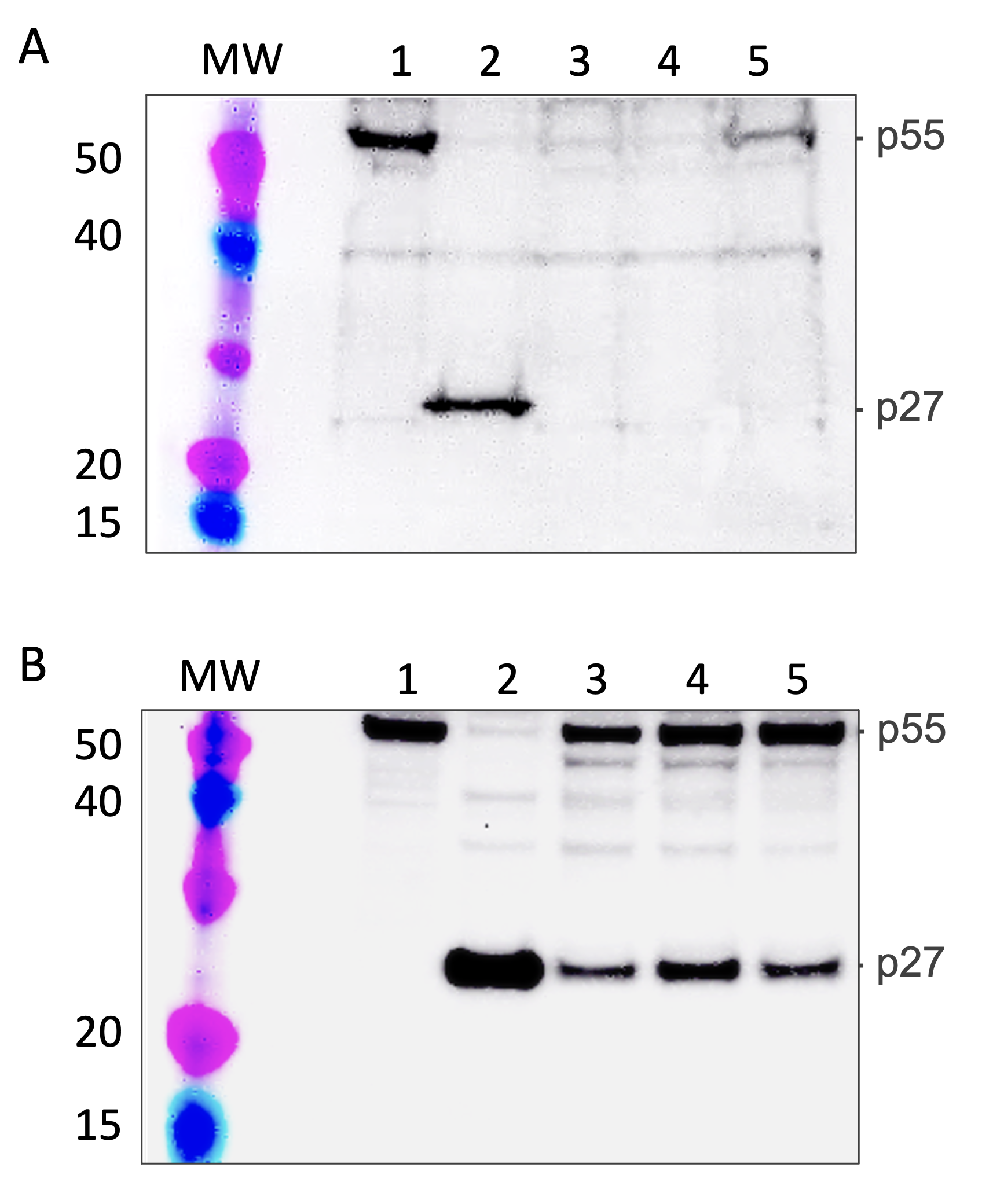
**

**Figure S3. Expression of SIV *gag-pol* mRNA constructs alone or in combination with *gag* mRNA.** (**A**) Immunoblot analysis showing the expression of uncleaved (p55) and protease-cleaved (p27) SIV Gag in 293T-WITO.2A11 cells transiently transfected with: 1. *gag* mRNA alone; 2. *gag + pro* mRNA; 3. *gag-pol* CO_hi_ mRNA; 4. *gag-pol* NF mRNA; 5. *gag-pol* NF_tr_ mRNA. The blot was developed using a rabbit polyclonal antiserum to SIV p27. (**B**) Western blot analysis showing the expression of uncleaved p55 and protease-cleaved p27 SIV Gag in 293T-WITO.2A11 cells transiently transfected with the same mRNAs as in (**A**) but with the addition of *gag* mRNA co-transfection in nos. 3, 4 and 5 (at 1:2 *gag-pol:gag* Wt:Wt ratio).

**SUPPLEMENTARY TABLES**

**Table S1. Area under the curve (AUC) values for HIV-1 trimer-binding and pseudovirus (PV) neutralizing antibody titers in serum of immunized mice.**

| **426c.ΔG3 trimer binding** | **Env** | **Env+Gag** | **Env+Gag +GagPol 1:5** | **Env+Gag +GagPol 1:10** | **Env+Gag +GagPol 1:20** |
| --- | --- | --- | --- | --- | --- |
| **Total AUC** | 8699 | 10277 | 13012 | 15004 | 16374 |
| **Standard Error** | 1076 | 1334 | 1881 | 1747 | 1824 |
| **95% CI** | 6591 to 10808 | 7663 to 12891 | 9325 to 16699 | 11579 to 18428 | 12799 to 19949 |
| **Peak Y** | 104 | 127 | 141 | 174 | 195 |
| Values are endpoint dilution titers x10^-3^ | |  |  |  |  |
|  |  |  |  |  |  |
| **426c.ΔG3 PV neutralization** | **Env** | **Env+Gag** | **Env+Gag +GagPol 1:5** | **Env+Gag +GagPol 1:10** | **Env+Gag +GagPol 1:20** |
| **Total AUC** | 45274 | 89157 | 221485 | 145719 | 92032 |
| **Standard Error** | 28494 | 40348 | 75500 | 53131 | 33886 |
| **95% CI** | 0 to 101122 | 10076 to 168238 | 73508 to 369463 | 41584 to 249854 | 25618 to 158447 |
| **Peak Y** | 16220 | 29838 | 71488 | 48337 | 21676 |
| Values are half-maximal neutralization titers (IC_50_) | | |  |  |  |
|  |  |  |  |  |  |
| **426c.ΔG1 PV neutralization** | **Env** | **Env+Gag** | **Env+Gag +GagPol 1:5** | **Env+Gag +GagPol 1:10** | **Env+Gag +GagPol 1:20** |
| **Total AUC** | 2029 | 2593 | 24888 | 5710 | 5705 |
| **Standard Error** | 941.2 | 997.4 | 11966 | 3052 | 2971 |
| **95% CI** | 184 to 3874 | 638 to 4547 | 1435 to 48343 | 0 to 11691 | 0 to 11529 |
| **Peak Y** | 648 | 707 | 7834 | 2671 | 1628 |
| Values are half-maximal neutralization titers (IC_50_) | | |  |  |  |

**Table S2. Area under the curve (AUC) values for SARS-CoV-2 spike trimer-binding and pseudovirus (PV) neutralizing antibody titers in serum of immunized mice.**

| **Wuhan-1 spike trimer binding** | **Spike-S** | **Spike-S+Gag** | **Spike-S+Gag +GagPol 1:5** | **Spike-S+Gag +GagPol 1:10** | **Spike-S+Gag +GagPol 1:20** |
| --- | --- | --- | --- | --- | --- |
| **Total AUC** | 10346 | 23484 | 23387 | 19505 | 16526 |
| **Standard Error** | 3266 | 9251 | 6162 | 5456 | 4728 |
| **95% CI** | 3945 to 16746 | 5353 to 41615 | 11309 to 35465 | 8811 to 30199 | 7259 to 25793 |
| **Peak Y** | 94 | 216 | 156 | 146 | 135 |
| Values are endpoint dilution titers x10^-3^ | | |  |  |  |
|  |  |  |  |  |  |
| **Wuhan-1 PV neutralization** | **Spike** | **Spike+Gag** | **Spike-S+Gag +GagPol 1:5** | **Spike-S+Gag +GagPol 1:10** | **Spike-S+Gag +GagPol 1:20** |
| **Total AUC** | 33734 | 55292 | 80437 | 88010 | 42510 |
| **Standard Error** | 17925 | 27295 | 20384 | 37112 | 13767 |
| **95% CI** | 0 to 68866 | 1795 to 108788 | 40485 to 120389 | 15272 to 160747 | 15527 to 69494 |
| **Peak Y** | 9301 | 15874 | 21793 | 21570 | 10319 |
| Values are half-maximal neutralization titers (IC_50_) | | |  |  |  |
|  |  |  |  |  |  |
| **B.1.351 PV neutralization** | **Spike** | **Spike+Gag** | **Spike-S+Gag +GagPol 1:5** | **Spike-S+Gag +GagPol 1:10** | **Spike-S+Gag +GagPol 1:20** |
| **Total AUC** | 36747 | 80961 | 95856 | 81984 | 59786 |
| **Standard Error** | 26601 | 40425 | 26918 | 45529 | 19407 |
| **95% CI** | 0 to 88883 | 1730 to 160192 | 43098 to 148613 | 0 to 171219 | 21749 to 97824 |
| **Peak Y** | 9981 | 19194 | 22779 | 20963 | 15429 |
| Values are half-maximal neutralization titers (IC_50_) | | |  |  |  |
